# Fruit Juice Industry Wastes as a Source of Bioactives

**DOI:** 10.1021/acs.jafc.2c00756

**Published:** 2022-05-11

**Authors:** Kevser Kandemir, Elif Piskin, Jianbo Xiao, Merve Tomas, Esra Capanoglu

**Affiliations:** † Faculty of Engineering and Natural Sciences, Food Engineering Department, Istanbul Sabahattin Zaim University, Halkali, 34303 Istanbul, Turkey; ‡ Department of Analytical Chemistry and Food Science, Faculty of Food Science and Technology, University of Vigo-Ourense Campus, E-32004 Ourense, Spain; § International Research Center for Food Nutrition and Safety, Jiangsu University, 212013 Zhenjiang, China; ∥ Department of Food Engineering, Faculty of Chemical and Metallurgical Engineering, Istanbul Technical University, 34469 Maslak, Istanbul, Turkey

**Keywords:** fruit juice, waste, bioactive compounds, phenolics, valorization

## Abstract

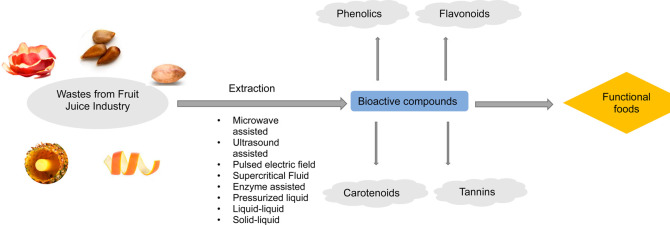

Food processing sustainability, as well as waste minimization, are key concerns for the modern food industry. A significant amount of waste is generated by the fruit juice industry each year. In addition to the economic losses caused by the removal of these wastes, its impact on the environment is undeniable. Therefore, researchers have focused on recovering the bioactive components from fruit juice processing, in which a great number of phytochemicals still exist in the agro-industrial wastes, to help minimize the waste burden as well as provide new sources of bioactive compounds, which are believed to be protective agents against certain diseases such as cardiovascular diseases, cancer, and diabetes. Although these wastes contain non-negligible amounts of bioactive compounds, information on the utilization of these byproducts in functional ingredient/food production and their impact on the sensory quality of food products is still scarce. In this regard, this review summarizes the most recent literature on bioactive compounds present in the wastes of apple, citrus fruits, berries, stoned fruits, melons, and tropical fruit juices, together with their extraction techniques and valorization approaches. Besides, on the one hand, examples of different current food applications with the use of these wastes are provided. On the other hand, the challenges with respect to economic, sensory, and safety issues are also discussed.

## 1 Introduction

Over the past few decades, the global demand for fruit production has increased due to the growing population and changing demographics to consume healthy foods. In this regard, acres gifted for fruit production have been steadily increasing throughout the world. It was estimated that over 640 million tons of fruits were harvested in 2011 and 870 million tons in 2018.^1,2^ Among all fruits produced, almost 50% of the crops are being processed as juice, and only from citrus juice production, annually 25 million tons of waste is being generated.^3^ These byproducts, which may account for 20–80% of the whole fruit, are practically important for environmental aspects; however, the ever-increasing interest in their valorization has emerged in new research areas.^3^ The huge volume of fruit wastes is now the concern for waste management challenges, especially when taking the number of malnourished people and natural source depletion into consideration. Therefore, waste minimization and valorization became an international debate that aim to enhance sustainability of the food industry.^4^ In the literature, various fruit wastes, including seeds, peels, pomace, stems, leaves, and stones were evaluated according to their chemical compositions, and remarkable amounts of bioactive components were identified.^5−8^ In conjunction with growing interests to find functional foods and healthier options, the food industry became more aware of the significance to find natural food additives to provide value-added end products with health-promoting effects.^9^ Therefore, the determination of bioactive components, specifically phenolics, has gained attention to obtain value-added byproducts from agro-industrial wastes.^10^ Phenolic compounds consist of an aromatic ring and one or more hydroxyl substitutes in their structures, and their ability to conjugate with mono- and polysaccharides allows their structural diversity, in which more than 8000 phenolics are identified in the nature, and more than 6000 phenolic compounds are flavonoids in plants.^11^ In addition to flavonoids including anthocyanidins such as pelargonidin, malvidin, cyanidin, and delphinidin, fruits and fruit wastes are also considered as rich sources of phenolic acids including gallic acid, ellagic acid, and vanillic acid.^12^ The importance of these compounds arises from their ability to act as a free radical scavenger or an antioxidant, which is triggered by hydrogen or an electron-donating ability that is affected by the number and the position of the hydroxyl groups present in their structure.^11^ Thanks to their antioxidant properties, they play an important role as being antidiabetic, antitumor, antihypertensive, anti-inflammatory, antiaging, cardioprotective, and neuroprotective agents.^13−16^ With this point of view, there are numerous studies that focus on the development of novel functional food products such as yoghurt and kefir as well as extruded snacks, short-dough biscuits, and functional cookies by fortification with fruit wastes.^17−20^ Therefore, recovery of phenolic compounds from byproducts of the fruit juice industry is a big deal, especially when it comes to find a sustainable and cost-effective source of the bioactive compounds, which could be incorporated into various food matrices to improve their nutritional value and also could be used as a natural food colorant.^21,22^ Although huge amounts of waste are being generated in the food industry, seasonal production and the variable composition of the waste products bring an important disadvantage for their industrial utilization. Therefore, it is recommended to solve this problem by encouraging pilot-scale on-site processing of the seasonal wastes from different industries.^23^

Although fruit wastes are eco-friendly, sustainable, and cost-effective sources, finding appropriate extraction methods to obtain a high extraction efficiency without further utilization of hazardous chemicals is as important as the source of bioactive compounds to improve the sustainability of the food system.^24^ However, novel or modified conventional extraction methods can contribute to the valorization of fruit waste bioactives in two ways, namely, (1) they may annihilate the disposal of toxic chemicals to the environment as a result of extraction processes and (2) they may enable maximal efficiency of extraction in a shorter period of time, which at the end leads to a reduction in the production costs. Conventional extraction methods are generally based on an organic solvent, often confronted as liquid–liquid or solid–liquid extraction methods. On the one hand, in addition to organic solvent usage, another disadvantage of these techniques is the incorporation of an evaporation step, which can not be ignored due to a high possibility of thermal destruction of bioactive components.^25^ On the other hand, novel “green” methods are recently emerged in the literature, which can be listed as microwave-assisted extraction (MAE), pulsed electric field (PEF), ultrasound-assisted extraction (UAE), supercritical fluid extraction (SFE), enzyme-assisted extraction (EAE), and pressurized liquid extraction (PLE).^26^ In spite of their eco-friendly operation mechanisms, these novel technologies are known for their high investment costs; however, a low operation cost and high yields can eliminate this major disadvantage. Regarding this, Pingret et al.^27^ examined the possible extraction of phenolic compounds from apple peels by using UAE, with a solvent–solute ratio of 1:500 in a pilot tank with 30 L capacity, with an ultrasound output at 25 kHz and 200 W. Results showed that the UAE method was 15% more effective when compared to conventional extraction methods. A similar study also pointed out that the scale-up had 7.5 times higher extraction efficiency compared to the lab scale.^28^ The lab-scale extraction method of MAE was also optimized and applied to the pilot scale, and it was concluded that the pilot-scale extraction provided a greater phenolic content since the pilot equipment contained a mechanical stirrer, which provides an increase in the diffusion rate.^29^ As for economic aspects, a study has investigated the economic outputs of UAE in contrast to agitation extraction regarding solvent concentration, operation time, temperature, and the ratio of solvent to feed. Even though UAE caused indistinctly higher manufacturing costs compared to the conventional method, the solvent volume to feed mass ratio exhibited a lower cost. Therefore, the UAE method is stated as an alternative method for the extraction of phenolic compounds at the industrial level.^30^ Despite the presence of promising studies in the literature, extraction methods of phenolic compounds still need optimization and verification. Some phenolic compounds are stable under harsh conditions; however, most of them are extremely unstable, being generally susceptible to oxidation as well as volatility and thermolability. Therefore, a proper extraction method should be chosen with utmost care to enable careful extraction with no chemical alteration.^31^ Since there is no standardized procedure for the recovery of phenolic compounds, there is still a need to establish an optimization process concerning the target compound’s characteristics, which are discussed in more detail in the sections below. Nevertheless, information on the economic impact for different industries in the literature is still scarce.

Fruit wastes cause a huge economic impact to business entities, from handling to discharging. Therefore, valorization of fruit wastes is not only important for decreasing the waste load in the landfills but also important for manufacturers to encourage the establishment of a sustainable economy. Only in China and the United States, it has been reported that fruits and vegetables generated 32 and 15 million tons of waste, respectively.^32^ A growing population and a vast amount of fruit consumption worldwide have led scientists and manufacturers toward a circular economy, which allows recycling and valorization of waste materials to be put back into the food supply chain.^33^ The economic value of global food waste is estimated as 1000 billion dollars, which may reach up to 2600 billion when we consider all the environmental costs as a result of this action.^34^ The World Food Program (WFP) states that 250 million people suffer from famishment, which is not consistent with the aim of “zero hunger” by 2030.^35^ If the industries become conscious about food waste valorization and more research is done for the feasibility of the valorization techniques, manufacturers will have the advantage of selling their byproducts, instead of undertaking their discharge costs, and lower their prices in consequence of saving money.

Even though various extraction methods are present for the recovery of bioactive compounds from fruit byproducts, these compounds are usually extremely unstable against temperature, pH variations, oxygen, and processing conditions. Indeed, because of several reasons including solubility, these compounds have limited bioavailability. In this sense, the food industry is constantly evolving into producing value-added products with natural ingredients, which are tuned to express their substantiality.^36^ Encapsulation is a method in which a sensitive compound is packed into a wall material to manipulate the main characteristics of the core material and to create an effective barrier against harsh environmental conditions, in order to be used in functional food production. This method allows one to enhance the bioavailability as well as control the release at the target environment, in addition to improving the shelf life of functional food products.^37^ Microencapsulation mainly depends on storing the bioactive compounds in a microscopic-sized coating material, which mainly consists of carbohydrates, hydrocolloids, proteins, and lipids.^38^ Various techniques can be implemented for entrapment, depending on the intention of final utilization, the material to be coated, and the wall material. Therefore, physical (freeze-drying, spray drying, extrusion, etc.), physicochemical (ionic gelation, coacervation, etc.) and chemical methods are widely used in the literature.^36^ These techniques improve the utilization potential of byproducts from various fruits for the production of functional foods, which is especially important in the perspective of a circular economy. Currently, different food formulations have been reported in the literature. For instance, Çam et al.^39^ have achieved a successful ice cream formulation with a pomegranate peel extract, microencapsulated by spray drying. Results showed that pomegranate peel extracts have improved antioxidant and α-glucosidase activity, with no astringent taste in the final product, thanks to encapsulation. They also reported a better storage stability of phenolics when they are microencapsulated. Lately, pomegranate peel extracts have been coated by orange juice byproducts as a “green” coating material. Similar to previous research, the stability of phenolic compounds has been significantly improved compared to the crude extract. When incorporated into cookies, the baking procedure caused a higher loss in crude phenolic extracts compared to the encapsulated samples.^40^ The heat stability of bioactive compounds has been improved by encapsulation methods in various research.^41^ Encapsulated extracts had also a higher acceptability for their odor and color parameters compared to cookies with crude extracts.^40^ Since flavors are volatile and sensitive to processing conditions, microencapsulation allows the release of the flavor under certain conditions.^42^ In comparison to microencapsulation, nanoencapsulation is known for their higher potential of delivering bioactive compounds to any part of the body with a higher precision, thanks to its smaller, nanoscaled particle size. Reducing the size of the bioactive compounds is generally responsible for enhanced solubility and bioavailability, therefore improving the functional value of the compounds.^43^ Recently, a positive effect on the bioaccessibility and antioxidant activity of food bioactives has been observed when food bioactives are delivered through a nanobased system.^44^ Considering these studies, it can be concluded that fruit byproducts may be useful for improving the functional value of food products, if they are treated by a suitable method. Therefore, consumption of enriched food products may have a huge potential in preventing malnutrition-caused diseases and health problems. On the one hand, since the extraction yields are relatively low for fruit and vegetable byproducts, most researchers have not focused on implementing encapsulation procedures. On the other hand, encapsulation methods are not specific for the core material or processing conditions; therefore, there is still an interrogation mark on the method that is the most suitable for the intended use. Additionally, safety assays should be performed in vivo for novel food ingredients as well as for the establishment of the legal regulations for the protection of consumer rights and public health safety.

Although food valorization has advantages for a number of points described above, there are still gaps to be filled in the literature. For instance, different processing conditions and extraction techniques may yield different amounts and profiles of bioactives, depending on their type. Both novel and conventional methods have their own advantages and disadvantages; therefore, the method should be selected and implemented wisely by taking a food matrix and target compound into consideration. If necessary, the combination of different techniques and pretreatments can be useful to increase the extraction yield.^45^ Furthermore, the production of novel food ingredients at larger scales might be compelling in the technological and economical point of view. Lastly, a specific regulation should be legislated for the safety assessments of the bioactives from byproducts to prevent valorization of potentially hazardous compounds.^46,4600^

It is important to acquire knowledge on the types and contents of phenolic compounds in fruits and their wastes individually in order to valorize them efficiently. With a thorough understanding of these sources, it would be feasible to learn more about their potential for usage and develop the appropriate applications. Furthermore, along with the characterization of the bioactives in each fruit, especially phenolics, more information will be available about which types of waste can be used for which biological activity or health effect, the most accurate extraction conditions and methods for these, and which food/nutraceutical/pharmaceutical application would be the best for them. In this regard, this review emphasizes the main byproducts that are considered as waste in different fruit juice industries and their composition regarding bioactive components, mainly phenolic compounds and a comparative evaluation of different extraction methods for the recovery of bioactive compounds and their valorization. Current reviews in the literature mainly focus on some specific types of bioactive compounds present in the wastes of some specific fruits, and to the best of our knowledge there is no review paper covering different types of fruit juice wastes in such a broad range. In this context, we aim to provide a comprehensive review including all the relevant information and a guide for future studies.

## 2 Bioactive Compounds from Fruit Juice Processing Wastes

The fruit juice industry is one of the largest agro-based industries worldwide.^47^ Several fruits such as apples, oranges, peaches, and pineapples are used to produce fruit juices, which creates a considerable amount of waste.^48^ As stated before, peels, seed fractions, pulp, pomace, stem, and stones are regarded as different forms of fruit wastes.^49^ These fruit wastes have a large number of valuable components, namely, bioactive compounds, and have numerous beneficial effects on human health such as antioxidant, anti-inflammatory, and anticancer activities.^50^ Therefore, valorization of these wastes is of great importance. This section summarizes studies on bioactive substances obtained from fruit juice wastes and provides significant information for future studies. The main bioactive compounds in the wastes of different fruits and the important results obtained from different studies are summarized in Table 1 and Table 2.

**Table 1:** Main Bioactive Compounds in Fruit Wastes

fruit	waste	bioactives	refs
Apple	Peel	Anthocyanins, flavan-3-ols, dihydrochalcones, chlorogenic acid, procyanidin B2, epicatechin, caffeic acid, p-coumaric acid, ferulic acid	(72, 82, 302)
	Seed	Chlorogenic acid, protocatechuic acid, coumaric acid, caffeic acid, ferulic acid, *p*-coumaroylquinic acid, phloretin-2′-xyloglucoside, quercetin derivatives, (+)-catechin, (−)-epicatechin, phloridzin, proanthocyanin B2	(7, 87, 90)
	Pomace	Epicatechin, caffeic acid, phloretin-2′-xyloglucoside, phloridzin, quercetin derivatives, ursolic and oleanolic acids	(64, 69)
Citrus fruits	Peel	Caffeic acid, *p*-coumaric acid, ferulic acid, sinapic acid, naringin, hesperidin, nobiletin, tangeretin	(119)
	Seed	Limonin, nomilin, obacunoic acid, ichangin, deoxylimonoic acid, nomilinic acid	(303)
Berries	Peel	(+)-Catechin, epicatechin, caftaric acid, gallic acid, gallotanins, anthocyanins	(10, 304)
	Seed	(+)-Catechin, epicatechin, gallic acid, vanillic acid, syringic acid, procyanidins	(10, 304, 305)
	Pomace	(+)-Catechin, epicatechin, hydroxybenzoic acid, hydroxycinnamic acid, vanillic acid, caftaric acid, chlorogenic acid, rutin, naringenin, anthocyanins	(10, 169, 306, 175)
Stone Fruits	Peel	β-Carotene, catechin, chlorogenic acid, cyanidin-3-galactoside, cyanidin-3-glucoside	(216, 225, 307, 204)
	Stone/Kernel	Gallic acid, vanillic acid, benzoic acid, phloridzin, quercetin derivatives, catechin, epicatechin, 5-caffeoylquinic acid, quercitrin, quercetin	(221, 227, 308, 212)
	Pomace	β-Carotene, neochlorogenic acid, cyanidin-3-glucosyl-rutinoside, catechin	(172, 230)
Melons	Peel	Gallic acid, catechin, ellagic acid, kaempherol, cinnamic acid, ferulic acid, chlorogenic acid, rutin	(255)
	Seed	Gallic acid, caffeic acid, rosmarinic acid, protocatechuic acid, vanillic acid derivatives, quercetin-3-rutinoside, ellagitanins, derivatives of syringic and ellagic acids	(245, 248)
Tropical Fruits	Peel	Ethyl gallate and penta-*O*-galloyl-glucoside, gallotannins, gallic acid, catechin, epicatechin, ferulic acid, punicalagin (in pomegranate)	(6, 266, 273, 286)
	Seed	Gallotannins, gallates, gallic acid, ellagic acid	(266, 309, 310)
	Pomace	Quinic acid, caffeic acid, kaempferol derivatives, quercetin derivatives. catechin, chlorogenic acid, *p*-coumaric acid, protocatechuic acid	(277, 279)
	Core and Crown (pineapple)	Bromelain	(274, 275)

**Table 2:** Extraction Method and Main Results of the Studies on Bioactive Compounds of Different Fruit Wastes

	type of fruit	part	bioactivity parameter	extraction method	key points	refs
biactives in apple wastes	Gala apple	pomace	phenolics	not stated	Apple pomace polyphenols had strong antioxidant activities compared to vitamins C and E.	(67)
	Golden delicious and Panaia-Red	pomace	total phenols and flavonoids	solvent extraction	In golden delicious variety; contents of total phenols, total flavonoids and flavan-3-ols were found to be 6.8 ± 1.2 mg GAE/ 100 g dw, 3.8 ± 0.6 mg CE/100 g dw, and 0.6 ± 0.4 mg CE/100 g dw, respectively. In Panaia-red variety; contents of total phenols, total flavonoids and flavan-3-ols were found to be 15.5 ± 3.2 GAE/ 100 g dw, 11.0 ± 3.1 mg CE/100 g dw, and 7.5 ± 2.3 mg CE/100 g dw, respectively.	(5)
	Idared and Northern Spy	skin	phenolics	ultrasound assisted extraction	The apple skin of two cultivars from juice industry had 7-fold higher total phenolic content when compared with the pomace.	(80)
	Red Delicious	skin	phenolics and flavonoids	solvent extraction	The sample having skin represented higher phenolic and flavonoid contents.	(79)
	Chinese Red-Fleshed	peel, flesh, fruit	total polyphenols, total flavonoids, anthocyanins	solvent extraction	The apple peel had the highest total phenolic content (5429.92 ± 293.05 mg GAE/kg). The highest flavonoid content was measured in the peels (1544.40 ± 45.45 mg/kg), followed by whole apples (1266.86 ± 45.44 mg/kg) and flesh (1156.62 ± 5.12 mg/kg). The peel had the highest content of anthocyanins (195.45 ± 12.36 mg/kg).	(311)
	Gale Gala, Starking, Honeycrisp, Fuji, Qinguan, Golden Delicious, and Qinyang	seed, skin and flesh	antioxidant activity	ultrasound assisted extraction	Apple seeds had higher antioxidant activity than skins or flesh. It was in line with the phenolic content.	(7)
bioactives in citrus wastes	Criolla orange, Oneco tangerine, Tangerine-lemon, Sour orange and Valencia orange	seed	limonoids	ultrasound assisted extraction	The highest amount of limonoids were found in Oneco tangerine seeds as 0.75% per total dry weight.	(120)
	Dao lime, Vinh orange and Thanh Tra pomelo	seed	limonoids	solvent extraction	The highest limonoid content was reported as 41.73 μg/g in Pomelo by methanol extraction.	(126)
	Mandarin (*Citrus unshia* Marc. Var. *Kuno*)	peel	flavonoids	supercritical extraction	Hesperidin was found as the most abundant flavanone, as well as narirutin, rutin, and chlorogenic acid.	(312)
	*Citrus aurantofolia*	pomace	phenolic compounds, total phenolic and flavonoid contents	solvent extraction	Trans-ferulic acid was the major phenolic found in the extracts (2.86 mg/g), followed by hesperidin and chlorogenic acid. Total phenolic content ranged from 94.95 to 130.85 mg GAE/g.	(313)
	Satsuma mandarin, Femminello Comune lemon, Valencia orange and Fantastico bergamot	pomace	phenolic compounds and carotenoids	solvent extraction	Orange and lemon contained ferulic acid as a phenolic acid, whereas mandarin and bergamot contained vanillic acid. Bergamot had the lowest phenolic content. In all samples, hesperidin was the most abundant phenolic, followed by narirutin and eriocitrin. Mandarin and orange had the highest amount of carotenoids.	(314)
	*Citrus sinensis* (Orange), *Citrus reticulata Blanco* (Mandarin), *Citrus limon* (Lemon), *Citrus aurantifolia* (Key lime), *Citrus maxima* (Pomelo), *Citrus paradisi* (Red/Yellow/Green grapefruit).	pulp and peel	phenolic compounds	solvent extraction	For all fruits, chlorogenic acid was the predominant phenolic acid, whereas gallic acid was the lowest. Peel of the fruits was richer in tannins and total phenolics compared to the pulp.	(315)
	Clementine mandarins, Satsuma mandarins, navel orange, common orange	peel	flavonoids, carotenoids, vitamin C	solvent extraction	Owari had the highest amount of flavanone glycosides (hesperidin) and carotenoid content (β-cryptoxanthin) with 55.82 mg/g and 1278 μg/100 g, respectively. All varieties showed similar vitamin C contents.	(128)
	Clementine mandarin, Satsume mandarin, Hybrid mandarin, Navel orange, Common orange and Pigmented orange	pulp	narirutin, hesperidin, vitamin C	solvent extraction	Pigmented orange contained the highest amount of vitamin C (46 mg/100 g fw), whereas navels were high in hesperidin (73.8 mg/100 g fw) and satsumes in narirutin (27.6 mg/100 g fw).	(127)
	Orlando orange, Kinnow mandarin, Eureka lemon	peel and pulp	phenolics, vitamin C	solvent extraction	Orlando orange peel was the richest by means of phenolics (178 mg GAE/100 g dw). Only lemon pulp had more phenolics than its peel. All cultivars had similar ascorbic acid contents.	(129)
	*Citrus reticulata* (kinnow mandarin)	peel	total phenolic content	ultrasound-assisted extraction	Total phenolic content of kinnow mandarin was found as 36.17 mg GAE/g dw.	(316)
	*Citrus sinensis* (orange)	pomace	total phenolic content	ultrasound-assisted extraction	Total phenolic content of orange was reported as 91.96 mg GAE/g.	(317)
	Grapefruit, Sweet orange, Lemon	peel	phenolics	solvent extraction	For all fruits, total polyphenol contents were higher in their peels compared to their pulp.	(130)
	*Citrus unshiu*	peel and pulp	total phenolic and flavonoid contents	solvent extraction	Naringin and neohesperidin were only found in citrus peel. Compared to pulp, peel of the citrus fruit was found to be 5 and 2.3 times richer source of total phenolics and flavonoids, respectively.	(116)
bioactives in berry wastes	Grape (*Vitis vinifera L.*)	pomace	total phenolic content, total monomeric anthocyanins	microwave and ultrasound-assisted extraction	The maximum values of total phenolic and total monomeric anthocyanins were obtained by microwave-assisted extraction as 6.68 mg GAE/g dw, and 1.32 mg malvidin-3,5-diglycoside/g dw, respectively.	(318)
	Chardonnay, Macabeu, Parellada, Premsal Blanc grapes	pomace and stem	total phenolic and proanthocyanidin contents	accelerated solvent extraction	Parellada cultivar yielded the greatest amount of TP (4654 mg GAE/100 g dw) and proanthocyanidins (92.1 mg tannin/g dw).	(144)
	Merlot grape	pomace, seed and skin	total phenolic, tannin and anthocyanin content	solid–liquid extraction	Total phenolics were 59.6 mg GAE/g, total anthocyanin content was 0.53 mg/g in grape juice pomace.	(10)
	Raspberry (*Rubus idaeus* L., Rosaceae)	pomace	total phenolic content, total flavonoid content, total anthocyanin content	ultrasound-assisted extraction	Total phenolic content, total flavonoid content and total anthocyanin content of raspberry were found as 27.79 mg GAE/L extract, 8.02 mg QE/g pomace and 7.13 mg cyanidin-3-glucoside/L extract, respectively.	(319)
	Strawberry (*Fragaria × ananassa* Duchesne)	pomace	anthocyanin content	solid–liquid extraction	15 compounds were identified in strawberry and the most abundant ones were quercetin-3-glucuronide, kaempferol-3-glucuronide, tiliroside, ellagic, malic, succinic, citric and *p*-coumaric acid.	(320)
	Strawberry	pomace	anthocyanins and proanthocyanidins	ultrasound assisted extraction	Most abundant anthocyanins were found to be pelargonidin-3-glucoside, cyanidin-3-glucoside, 3-malonyl glucoside, and 3-rutinoside. Strawberry pulps contained high amounts of condensed tannins (up to 163 mg/100 g raw strawberry).	(157)
	Blackcurrant	pomace	total phenolics and total anthocyanins	solvent extraction	Blackcurrant extract contained 66.8 g/100 g total phenolics and 48.9 g/100 g total anthocyanins. Myricetin, quercetin, kaempferol and their glycosides were reported as the dominant flavonol aglycones and glycosides.	(166)
	Blackcurrant	pomace	total phenolics and total flavonoids	ultrasound assisted extraction	Chlorogenic acid was found to be the predominant phenolic, followed by rutin, naringenin and caffeic acid.	(169)
	Blackberry (*Rubus fruticosus L.*)	pomace	polyphenolic compound profile, total phenolic and ascorbic acid contents	maceration solvent extraction	Total phenolic content of blackberry was reported as 4618 mg GAE/100 g dw and l-ascorbic acid was 39.08 mg/100 g dw Blackberry pomace contained high amounts of pyrocatechol, followed by catechin and p-coumaric acid.	(176)
	Blackberry	pomace	total phenolic content, total monomeric anthocyanins and individual phenolic compounds	acid hydrolysis	Total phenolic content of blackberry was reported as 4016.43 mg GAE/100 g dw. Caffeic acid was the major phenolic acid. High amount of quercetin and cyanidin-3-glucoside were identified.	(174)
	Blueberry, red raspberry, blackberry and red currant	pomace	total phenolic content and anthocyanin profile	solvent extraction	11 different anthocyanins were observed in blueberry pomace, however, other berries contained only cyanidin derivatives, where cyanidin-3-*O*-glycoside existed in all pomace samples. Cyanidin-3-*O*-sophoroside was a distinctive anthocyanin for red raspberries. Red currants had the highest total phenolic content.	(175)
	Chokeberry (*Aronia melancocarpa*)	pomace	anthocyanin content	solvent extraction	Most aboundant anthocyanin in chokeberry was reported as cyanidin-3-*O*-galactoside (62%), followed by cyanidin-3-*O*-arabinoside (30%), cyanidin-3-*O*-xyloside (4%), and cyanidin-3-*O*-glucoside (2%). Total anthocyanin content was 62.8 mg/g dw.	(321)
	Cranberry	pomace	total phenolic, total anthocyanin content and anthocyanin profile	pressurized ethanol extraction	8.42 mg cyanidin-3-glucoside dw anthocyanins were determined, major ones being cyanidin-3-galactoside, cyanidin-3-arabinoside, peonidin-3-galactoside, and peonidin-3-arabinoside. Total phenolic content was reported as 84.96 mg GAE/g dw.	(322)
	Rowanberry (*Sorbus aucuparia L.*)	pomace	total carotenoid and β-carotene contents	supercritical extraction	Total carotenoid and β-carotene contents were reported as 78.91 mg/100 g dw and 38.69 mg/100 g dw, respectively.	(323)
	Raspberries, black currants, red currants, white currants, white and red gooseberries, blackberry, goji, and three Cv. (Duke, Blue Ray, and Misty) of blueberries	extract	phenolic compounds	solvent extraction	Results showed that black currants, blackberries, and blueberries had the highest total anthocyanin and total phenolic contents.	(324)
	Black currant, blueberry, raspberry, red currant, and cranberry	extract	anthocyanin profile	solvent extraction	Malvinidin-3-*O*-galactoside and malvinidin-3-*O*-arabinoside were reported as the most abundant anthocyanins among other 15 anthocyanins identified.	(185)
bioactives in stone fruit wastes	Apricot	peel and pulp	carotenoids	solvent extraction	Apricot peel had carotenoids 2–3 times higher than its pulp.	(204)
	Apricot	peel and pulp	phenolics	ultrasound assisted extraction	Phenolic compounds in the apricot peel to be ∼2–4 times higher than the pulp.	(206)
	Apricot	pomace	phenolics	ultrasound assisted extraction	The total phenolic content of the apricot pomace extract was 15.43 ± 0.03 mgGAE/ g dw of the extract, and the total flavonoid content was 11.9 ± 0.10 mg QCE/g dw of the extract.	(169)
	Peach	peel and pulp	catechin and chlorogenic acid	solvent extraction	Catechin and chlorogenic acid was higher in the peel than the pulp (catechin in the pulp: 1160 mg/kg, chlorogenic acid in the pulp: 2147 mg/kg; catechin in the peel: 1342 mg/kg, chlorogenic acid in the peel: 4578 mg/kg).	(216)
	Peach	peel and pulp and seed	polyphenols, flavonoids and carotenoids	solvent extraction	Peach pulp contained more polyphenols and flavonoids than the peel. In addition, carotenoid concentration of peach peel was approximately 6 times higher compared to the seed and pulp.	(218)
	Peach	kernel	polyphenols, carotenoids and tetraterpenoids	assisted extraction	Total polyphenol range was 3.8–12.7 g/100 g dw, while carotenoids ranged between 0.0–101.7/100 g dw and cyanogenic glycoside range was 17.4–245.7 mg/100 g dw.	(221)
	Peach	kernel and fruit	antioxidant activity and total phenol content	solvent extraction	Antioxidant activity (IC_50_) of peach extract was 2.66 μg/ mL, while it was 7.88 μg/ mL in peach kernel extract. The total phenolic content in peach was 253.4 mg GAE/100 g, while it was 29.3 mg GAE/100 g in peach kernel.	(325)
						
	Plum	peel and fruit	anthocyanin	solvent extraction	The most abundant anthocyanins were cyanidin-3-galactoside and cyanidin-3-glucoside. In addition, myrobalan plums had more anthocyanin and phenolic contents and higher antioxidant activity than red and yellow types.	(225)
	Sour cherry	pomace	total phenolic content	solvent extraction	Total phenolic content of sour cherry pomace was 91.29 mg GAE/g dw.	(229)
	Sour cherry	pomace	total anthocyanins, total phenolics and antioxidant activity	conventional and ultrasonic extraction	Total anthocyanins (mg/L) was 35.08 ± 1.06 - 38.20 ± 1.20, while total phenolics (mg/L) was 453.27 ± 7.42 - 493.84 ± 5.12 and antioxidant activity (mM Trolox/mL) was 59.61 ± 1.24 - 106.80 ± 0.71.	(231)
bioactives in melon wastes	Mazooun melon	seed	total phenolic content and phenolic profile	solvent extraction	Total phenolic content of 304 mg/100 g was determined. The most abundant phenolic acid was gallic acid, followed by caffeic acid, rosmarinic acid and protocatechuic acid.	(245)
	Cantaloupe	seed	total phenolic content	solvent extraction	Total phenolic content of cantoloupe seeds was 285 mg GAE/100 g extract. The highest phenolic content was found in cantaloupe leaf, followed by stem, skin, seed and flesh.	(247)
	Honeydew	seed	total phenolic content and phenolic profile	solvent extraction	Total phenolic content was 81.2 mg/100 g. Nine phenolics were identified, including caffeic acid, vanillic acid derivatives, quercetin-3-rutinoside, ellagitanins, derivatives of syringic and ellagic acids.	(248)
	Melon (*Cucumis melo L.*)	peel and seed	total phenolic content	enzyme-assisted solvent extraction	Total phenolic content of melon peel ranged between 35.03 to 222.62 mg GAE/g dw and 19.75 to 86.42 mg GAE/g dw in melon seeds.	(326)
	Galia melon	seed	total phenolic content	solvent extraction	Total phenolic content of galia melon seed was reported as 57.2 mg catechin/100 g dw.	(249)
	Watermelon	seed	total phenolic content	solvent extraction	Total phenolic content of watermelon seed was reported as 969.3 mg catechin/100 g dw.	(249)
	Piel de Sapo melon	seeds, juice, peel and pulp	phenolics, carotenoids and vitamin C content	solvent extraction	Peels and seeds contained similar amounts of phenolics and they presented the greatest values. They also found similar values of vitamin C for juice, pulp and peel, where seeds had lower values. No carotenoid was identified.	(253)
	Cantaloupe	pulp and peel	total phenolic content and carotenoids	solvent extraction	Cantaloupe melon contained 68.92 mg/g carotenoids in its pulp. Peel itself contained 31% of the total phenolic content, which is comparable with the phenolics in juice (35%).	(244)
	Watermelon	peel	total phenolic content and phenolic profile	solvent extraction	Total phenolic content was found as 335 mg catechin/100 g dw, and mainly contained gallic acid, catechin, ellagic acid and kaempferol.	(249)
bioactives in tropical fruit wastes	Mango	peel	antioxidant activity	solvent extraction	Gallate and penta-*O*-galloyl-glucoside, from mango peels presented potent hydroxyl radical, superoxide anion and singlet oxygen scavenging activities.	(6)
	Mango	peel	total phenolics, flavonoids, and carotenoids	solvent extraction	Total phenolic range was 2930 to 6624 mg GAE/100 dw while the flavonoid range was 502–795 mg CE/100 g dw, and the carotenoid range was 3.7–5.7 mg/100 g dw.	(327)
	Mango	seed	phenolics	solvent extraction	Extracts of mango seed kernel had phenolic components with high antioxidant activity as well as tyrosinase inhibitory activity. The extracts also had high metal-chelating, radical scavenging and tyrosinase inhibitory activities.	(265)
	Mango	peel and kernel	gallotanins	solvent extraction	Gallotannins in kernel was approximately 4 times higher than in the peel (peel: 4 mg/g dw, kernel: 15.5 mg/g dw).	(266)
	Mango	peel and kernel	phenolics, ascorbic acid and carotenoids	solvent extraction	Total phenolics, ascorbic acid and carotenoid contents in dried mango peels were higher compared to the kernel.	(269)
	Pineapple	peel	phenolics	solvent extraction	Phenolic antioxidants in pineapple peel was found as 2.01 mmol/100 g fw.	(272)
	Pineapple	core	vitamin C and β-carotene	solvent extraction	Pineapple core had ∼2 times higher vitamin C content, while the rind had ∼2.5 times higher β-carotene.	(270)
	Kiwi	pomace (without peel)	total phenolics	solvent extraction	Total phenolics in kiwi pomace: 421 ± 12 mg GAE/g dw. Anthocyanins and flavan-3-ol were not determined.	(277)
	Kiwi	pomace	total polyphenols and antioxidant capacity	microwave-assisted extraction	At the optimal conditions of microwave-assisted extraction (*T*: 75 °C; time: 15 min, solvent composition: 50% ethanol:water, and solid-solvent ratio: 1:15), total phenolic content of 4.79 ± 0.13 mg GAE/g dw was obtained from the kiwi pomace. In addition, the antioxidant capacity, measured with the DPPH and ABTS assays, of the optimized extracts were EC_50_ = 5.49 ± 0.02 mg and 560 ± 1 μg, respectively.	(328)
	Kiwi	seed	antioxidant activity	solvent extraction	DPPH antioxidant activity of seeds of different species of kiwi fruit was reported to change from 25.7 to 35.1 IC_50_ mg/mL.	(281)
	Pomegranate	peel and seed	total antioxidant capacity	solvent extraction	Peels represented high total antioxidant capacity while seeds had a lower value.	(287)
	Pomegranate	peel and seed	total phenols, proanthocyanidins and antioxidant activity	homogenizer-assisted extraction and Soxhlet ethanol extraction	Total phenolics in peels and seeds were 24.1 mg/g and 20.2 mg/g, respectively. Proanthocyanidins in peels and seeds were 3.1 mg/g , 1.6 mg/g, respectively. Antioxidant activities (% inhibition) of peels and seeds were 91%, 18%, respectively.	(329)
	Pomegranate	peel, pulp, and seeds	antioxidant activity	solvent extraction	Peel showed the highest antioxidant activity.	(288)

### 2.1 Apple

Apples and apple products are extensively consumed across the world.^51,52^ According to Food and Agriculture Organization (FAO) statistics, apples are grown worldwide, and in 2020, its global production exceeded 86 million tons in estimated 4.6 million hectares area.^2^ While the majority of apples are consumed as fresh, 25–30% is used for the production of processed products, with apple juice concentrate (65%) as the main product.^53^ Apple juice is the second most popular juice after orange juice worldwide.^54^ Significant amounts of waste are produced by apple juice processing, accounting for 25–40% of the mass of processed apples depending on the method used for juice extraction.^55^ The residues from apple juice processing include mainly peels, seeds, and pulp, known as apple pomace.^56^ Essentially, apple pomace is the main waste of the apple extraction process, which represents up to 30% of the original fruit.^51^ It is composed of water (76.3%) and dry solids (23.7%) and includes apple skin/flesh (95%), seeds (2%–4%), and stems (1%).^53,57^

Apple pomace is a source of phytochemicals and bioactive compounds, such as polyphenols, dietary fibers, triterpenoids, and volatiles.^58^ Essentially, it is a good source of polyphenols, because it mainly contains the skin of the fruit with phenolics.^59,60^ The composition and concentration of phenolic compounds in the peel and flesh of the apple varies, with the peel having a higher concentration than the flesh.^61−63^ The major polyphenols in apple pomace was reported as epicatechin, caffeic acid, phloretin-2′-xyloglucoside, phloridzin, and quercetin derivatives.^64^ Apple pomace has been investigated as a promising source of bioactive polyphenols, owing to the growing interest in natural sources of antioxidant compounds.^65^ Because of their antioxidant and antimicrobial properties, many potential applications are available for these bioactive polyphenols in food, pharmaceutical, and cosmetic industries.^66^ Lu and Foo (2000) studied the antioxidant properties of apple pomace polyphenols, which have been shown to have strong antioxidant activities compared to antioxidant vitamins C and E.^67^ Lu and Foo (1997) reported that more than half of the polyphenols in apple pomace are quercetin glycosides.^64^ Maragò et al.^5^ measured the level of phenolics in apple juice and its byproduct (apple pomace) in two different apple varieties as Panaia red and Golden delicious. On the one hand, the concentrations of total phenols (TP), total flavonoids, and flavan-3-ols in Golden delicious were 6.8 ± 1.2 mg of gallic acid equivalents (GAE) per 100 g of dw, 3.8 ± 0.6 mg catechin equivalent (CE)/ 100 g dw, and 2.6 ± 0.4 mg CE/ 100 g dw, respectively. On the other hand, these values were 15.5 ± 3.2 GAE/ 100 g dw, 11.0 ± 3.1 mg CE/ 100 g dw, and 7.5 ± 2.3 mg CE/ 100 g dw, respectively, for the Panaia red variety, demonstrating that Panaia red is much richer in phenolics compared to Golden delicious. The important biomolecules from apple pomace also include triterpenoids, which are secondary metabolites synthesized from isopentenyl pyrophosphate oligomers,^68^ which present six isoprene units (C_5_H_8_).^56^ Triterpene acids are found in apples, particularly in the cuticular wax of the peels, with ursolic and oleanolic acids being the most abundant.^69^ Pentacyclic triterpenes such as ursolic acid have gained much attention from researchers due to their antioxidant, antitumor, anti-inflammatory, and antibacterial properties.^70^ Pentacyclic triterpenoids, in addition to their biological activity, have no obvious toxicity and are therefore potential chemicals to be used in new medicinal products.^71^

Apple peels contain anthocyanins, flavonols, flavan-3-ols, dihydrochalcones, and phenolic acids, which have been correlated with health-promoting benefits.^72^ In fact, an apple peel is mainly rich in phenolic compounds compared to other apple byproducts due to their physiological function in protecting the fruit against ultraviolet radiation.^73^ This is supported by several studies showing that apple peel may have greater antioxidants and antioxidant capacity than the pulp fraction or the whole fruit.^74−78^ Eberhardt et al.^79^ reported the contents of phenolics and flavonoids in apple with skin as 290 mg/100 g apple and 142.7 mg/100 g apple, respectively; however, these values were 219.9 mg of phenolics and 97.6 mg of flavonoids per 100 g of apples without skin. In another study conducted by Rupasinghe and Kean,^80^ the apple skin of two cultivars (Idared and Northern Spy) from the juice industry was reported to have over sevenfold higher total phenolic content when compared with the pomace. In addition, the skin of Idared apple was shown to have fivefold greater levels of total phenolic compounds. Moreover, with regard to polyphenols, quercetin-3-*O*-galactoside was predominant, followed by quercetin-3-*O*-rhamnoside and quercetin-3-*O*-glucoside in the skin of two cultivars. Sun-Waterhouse et al.^81^ measured and compared the bioactive content and appearance attributes of juices and skin wastes of three apple genotypes including white fleshed (WF), pink fleshed (PF), and red fleshed (RF). The total extractable phenolic content of RF apple was shown to be the highest, while PF had the least.^81^ Lončarić et al.^82^ recovered polyphenols from the peel of 12 traditional and 8 commercial apple varieties by the Micro-Matrix Solid-Phase Dispersion extraction method. They reported that the peel of traditional apples had higher contents of all investigated polyphenols. They also calculated the relative contribution of polyphenol groups and reported the major contributors to the total polyphenolic content in traditional and commercial apple varieties as nonflavonoids (28.6%) and flavanols (46.2%), respectively. In addition to these, chlorogenic acid, procyanidin B2, and epicatechin were reported to be the most abundant polyphenols in traditional apple peel, while procyanidin B2 was reported in commercial apple varieties.^82^

Apple seeds represent high oil content, and oleic acid and linoleic acid were reported to be the main fatty acids.^83−85^ A high percentage of unsaturated fatty acids makes apple seed oil nutritionally favorable, having positive effects on lowering low-density lipoprotein (LDL) cholesterol and preventing risks of cardiovascular diseases.^83^ In addition to fatty acids, apple seed also contains a high proportion of polyphenols similar to other apple juice industry byproducts. Phloridzin was reported by several researchers to be the major polyphenol in apple seeds.^86−90^*p*-Coumaroylquinic acid, phloretin-2′-xyloglucoside, (−) epicatechin, and chlorogenic acid have been also reported in apple seed extracts.^90^ Xu et al.^7^ reported apple seeds to have higher antioxidant activity than peels or flesh. In addition, a correlation analysis revealed that the total phenolic content (TPC) was correlated with three antioxidant activity assays, namely, ferric reducing antioxidant power assay (FRAP), 2,2-diphenyl-1-picrylhydrazyl assay (DPPH), and 2,2′-azino-bis-3-ethylbenzothiazoline-6-sulfonic acid assay (ABTS). Besides the phenolic compounds, apple seeds also accumulate cyanogenic glycosides, which are produced by various plant species as a secondary metabolite of nitrogen metabolism.^91,92^ It is known that cyanogenic glycosides are toxic, since they release the cyanide in their composition as HCN.^93^ Therefore, consumption of cyanogenic glycosides found in various fruit seeds can cause acute or subacute cyanide poisoning.^94^ Humans can develop acute cyanide poisoning at dosages ranging from 0.5 to 3.5 mg/kg body weight.^95^ The most abundant cyanogenic glycoside in fruits and fruit seeds and kernels is amygdalin.^96^ In a study examining the amygdalin content of apple seeds, it was determined that a total of 15 types of apple seeds contain amygdalin in amounts ranging from 1 to 4 mg/g.^94^ In this context, although a single apple seed does not cause any adverse effects when consumed, heavy consumption may cause poisoning. Since apple seed extract may contain high amounts of amygdalin, it may be necessary to remove this substance. Solid-phase extraction using a C_18_ extraction column is reported as one of the widely used extraction methods for amygdalin.^97^ In another perspective, amygdalin is reported in several researches as an active ingredient for the treatment of some health disorders such as asthma,^98^ atherosclerosis,^99^ diabetes,^100^ and tumors.^101^ However, the issue of whether amygdalin has therapeutic properties has not yet been sufficiently clarified. Discussions and research on the subject still continue, and therefore further research is required.

Extraction is the most critical procedure in recovering bioactive compounds from fruit wastes. The extraction technique employed varies depending on the target compound, and the appropriateness of the method used has a huge impact on the quantities of the extracted compound.^48^ Both conventional and novel extraction methods are applied to obtain bioactive compounds from apple byproducts.^102^ Among the conventional methods, maceration and Soxhlet extraction, which are performed at high temperatures using organic solvents such as aqueous acetone, methanol, and ethanol solutions at 60%–100% concentrations,^103^ are mostly used for comparison of alternative techniques or for the evaluation of some initial extraction parameters such as the mass ratio of the solvent and the sample.^102^ A comparative study in which four extraction methods, maceration, PLE, UAE, and MAE with different solvents was applied to apple pomaces from four different cultivars, was conducted.^104^ MAE with ethanol provided the highest efficiency in a short time and was reported to be the best for the extraction of antioxidants. Casazza et al.^105^ compared the conventional method, solid liquid extraction (SLE, 25 °C, 19 h), with nonconventional methods MAE (110 °C, 60 min) and High Pressure and Temperature Extraction (HTPE, 150 °C, 150 min) on the recovery of phenolic compounds from the peels of four apple and three grape cultivars. While HTPE provided the highest polyphenol extract in all apple varieties, similar yields were obtained in MAE and SLE. However, although similar results were obtained in these two processes, the fact that the MAE process takes 1 h and the SLE process takes 19 h makes MAE much more advantageous. Recently, Egüés et al.^106^ worked on the optimization of the extraction of antioxidant compounds from apple pomace using UAE. The maximum phenolic extraction yield was obtained at the conditions of 20 min, 90 °C, and 50% ultrasound amplitude. However, under these conditions, DPPH antioxidant capacity showed a decrease, which was most probably caused by the exposure of phenolics to high temperature. Brahmi et al.^107^ optimized the extraction of phenolic compounds present in apple peel and grape seed. Furthermore, to valorize the byproducts, they fortified yogurt with their powders. Ethanol and acetone were reported to be the solvents for the best extraction yield of phenolics from apple peel and grape seed in 60 and 90 min, respectively. In addition, sensory analysis results revealed that yogurt with grape seed powders was more preferable.

The valorization of apple byproducts is not sufficiently achieved, although a large quantity of apple waste is generated from the apple juice industry.^108^ Currently, the number of studies performed to evaluate these byproducts is increasing. Catană et al.^55^ obtained a functional product in powder form from apple waste generated in the apple juice industry. As expected, the obtained powders were good sources of polyphenols and had high antioxidant potential. In addition, minerals were abundant in the powder as well as dietary fibers, which could increase the fiber content of products such as those from a bakery and pastry. In another study, apple pomace was partially used instead of wheat flour (10% and 20% w/w) in biscuits.^17^ The fortified biscuits were then subjected to in vitro digestion to estimate the glycemic index. Additionally, sensory properties were evaluated. Apple pomace reduced the glycemic index of biscuits from 70, which is categorized as high glycemic index, to 65 and 60, which are considered as intermediate glycemic index. Furthermore, most characteristics, such as firmness, crispiness, sweetness, sourness, and shortbread flavor, were unaffected by the apple pomace substitution. However, the reformulated biscuits were scored significantly higher for baked flavor compared to the control, possibly due to the faster development of the Maillard reaction in the apple pomace-containing biscuits.

In short, the apple juice industry represents a high number of byproducts containing several bioactive components (mainly phenolics). Various fields such as medicine, cosmetics, and food industries benefit from these bioactive substances. Especially in the food sector, with the increasing awareness of sustainable food as well as the trend toward functional foods, fruit juice industry byproducts that provide various bioactive compounds have become a good source. Since extraction is the most important step in recovering bioactive compounds, it is critical to apply the most appropriate extraction technique for the target compound and further use. Therefore, optimization studies are important in terms of providing the best extraction yield and quality of the obtained product to be used for different purposes.

### 2.2 Orange and Other Citrus Fruits

Citrus fruits, which consist of 40 different species of the *Rutaceae* family, belong to the group of most cultivated crops in the world; therefore, it would not be wrong to say higher levels of production of citrus fruits leads to higher amounts of citrus juice production.^109,110^ Nearly 30% of citrus fruits are processed as fruit juice, which is extracted from the fruit, and the residual part (may reach up to 80%) is named as agro-industrial waste.^111−113^ It is estimated that 119.7 million tons of citrus waste is produced worldwide, and therefore valorization of these byproducts is significant from both economic and environmental aspects.^114^ In developing countries, citrus waste is often discarded in landfills and rivers, resulting in environmental and water pollution and, eventually, depletion of the dissolved oxygen ratio.^115^ In comparison with other fruits, the edible part of citrus fruits represents a relatively smaller portion; therefore, it is important to evaluate the potential valorization scenarios from these fruits. The main byproduct of citrus juice processing is citrus peel, which is a rich source of flavonoids such as hesperidin, naringin, and rutin, and could be potentially used as a health promoter in the nutraceutical industry.^116^ That brings the idea of importance for the companies to maximize their profit either by optimizing processing conditions or by recovering their wastes to reuse value-added compounds.^117^ Utilization of these residues has the advantage of being cheap and renewable sources that contain a wide range of bioactives including flavonoids, tocopherols, carotenoids, and phytosterols.^118,119^ Therefore, byproducts from citrus juice production are considered as a valuable source of bioactive compounds, which possess antioxidant, anticarcinogenic, antiproliferative, anti-inflammatory, and neuroprotective properties to be used in both the pharmaceutical sector and food industry.^117,118^

Various bioactives can be extracted from the pulp, peel, or seeds of citrus fruits. Talking about seeds, depending on the genotype and extraction procedures, a high amount of seed oil can be extracted from citrus fruits in which saturated, unsaturated, and omega fatty acids were indicated in the lipid section of the seeds.^118^ Besides fatty acids, citrus seeds were also reported as a good source of limonoids (limonin, nomilin, ubacunone, etc.), which are highly oxidized triterpenoids and classified either as aglycones or glycosides, and specific to citrus seeds.^120^ It was estimated that citrus processing industry wastes generate up to 15 000 tons of limonoids, especially limonoid glucoside, which is tasteless and presents no toxicity.^121^ There are various studies on the anticarcinogenic, antitumor, antibacterial, and antifungal activities of these bioactive substances.^122,123^ It was reported in earlier research that limonoid ingestion in mice has triggered glutathione S-transferase activity.^124^ Another study was performed by nine women and seven men between the ages of 19 and 51 years to analyze the metabolic fate of limonin glucoside. As a result, high doses of limonin was found in the plasma of the test subjects, which unearths that limonin has high bioavailability in humans.^121^ Currently, more than 50 different limonoids have been discovered from different *Citrus* species. Among all limonoids, the most abundant aglycones are limonin, followed by nomilin, and the most abundant glycone is known as limonin.^125^ Montoya et al.^120^ examined different citrus fruit seeds from Criolla orange, Oneco tangerine, tangerine-lemon, sour orange, and Valencia orange. According to the results, Oneco tangerine seeds are reported as the richest sources of limonoids, containing almost 7500 mg of limonoids per kg of seeds. In a similar study where the seeds of Dao lime, Vinh orange, and Thanh Tra pomelo fruits were investigated, pomelo seed extract exhibited greater limonoid content and antioxidant capacity when methanol extraction was used.^126^

Citrus peels are also rich in bioactive compounds, especially in phenolics such as caffeic acid, *p*-coumaric acid, ferulic acid, naringin, and tangeretin.^119^ Cano et al.^127^ examined different cultivars of citrus fruits such as Clementine mandarin, Satsume mandarin, Hybrid mandarin, Navel orange, Common orange, and Pigmented orange, extracted by dimethyl sulfoxide (DMSO) and methanol. Their results showed that pigmented orange contained the highest amount of vitamin C (46 mg/100 g fresh weight (fw)), whereas navels were high in hesperidin (73.8 mg/100 g fw), and satsumes had the greatest amount of narirutin (27.6 mg/100 g fw). Similarly, Bermejo et al.^128^ showed that, among different cultivars of citrus fruits such as Clementine mandarin, Satsume mandarin, Navel orange, and Common orange, Satsume mandarin had the highest amount of flavanone glycosides (hesperidin) and carotenoid content (β-cryptoxanthin) with 55.82 mg/g dw and 1278 μg/100 g dw, respectively. However, they concluded that the vitamin C content did not significantly change among different cultivars. Al-Juhaimi^129^ reported similar results with Orlando orange, Kinnow mandarine, and Eureka lemon by means of vitamin C content. Additionally, both pulp and peel of the citrus fruits were analyzed, and only Eureka lemon was found to contain more phenolics in its pulp compared to the peel, just like Gorinstein et al.^130^ reported that, compared to peeled fruits, peels of grapefruit, orange, and lemons contained higher amounts of polyphenols, 155, 179, and 190 mg/100 g fresh fruits, respectively. Similar results were also reported by Kim et al.^116^ They also concluded that peels of citrus fruits are rich in both dietary fibers and phenolic contents, and therefore they might be utilized conveniently in the food industry for different purposes.^116^

Conventional extraction methods as discussed above have the limitations of being time-consuming, toxicity arising from the solvents, and high maintenance costs, and therefore researchers have turned toward greener applications to terminate organic solvent use and decrease extraction time and cost as well as to increase extraction efficiency.^42^ As a novel, green extraction method, the ultrasonic extraction method is examined in different studies. Compared to other technologies such as microwave and supercritical fluid extraction, ultrasonic extraction is reported as being a cheaper and simpler method for utilization. On the one hand, Sun et al.^131^ analyzed *Citrus succosa* Hort peels and reported that, although ethanol provides a poor extraction yield for β-carotene under conventional extraction (CE), when it is used along with ultrasound extraction, higher extraction yields were obtained. On the other hand, dichloromethane caused the degradation of all *trans*-β-carotene under the ultrasound treatment. Another study, in which the peels of *C. sinensis* L. Osbeck were examined, showed that ionic salts (ILs), which are known for their tunable properties and biocompatibility, have a great potential for carotenoid extraction when assisted with ultrasound. Among four different ILs, methylimidazolium chloride was found to be the most efficient, leading a carotenoid content of 32.08 ± 2.05 μg/g dw.^132^ Lately, Saini et al.^133^ performed a study on the optimization of ultrasonic parameters for lutein extraction from *Citrus reticulata*, and they concluded that time, amplitude, and temperature have significantly affected the extraction yield. Ultrasonic extraction was also compared with the EAE method, and the results indicated that, although EAE provided an improved extraction yield, UAE recovered greater amounts of total phenolics and flavonoids compared to the conventional methods.^134^ However, a combined application of UAE and EAE further enhanced the total flavonoids and phenolic content.^135^ When MAE was compared with UAE, MAE resulted in higher phenolic acid recovery than UAE and CE.^136^ MAE also had the advantage of reducing energy consumption up to 27 times in comparison to the hydrodistillation method, which is a conventionally used technique for recovery.^137^ The SFE technique is responsible for higher yields of bioactive compounds by utilizing nontoxic carbon dioxide; however, lower diffusion rates cause longer extraction times of the bioactive compounds into the supercritical fluid.^138^ Lately, Priyadarshani et al.^139^ reported up to 93% lycopene yield by SFE from *Citrus paradisi* endocarp, depending on the pressure, extraction time, and their interaction.

Food fortification is described as an addition of micronutrients to various food matrices to improve their nutritional value, which is widely studied for citrus byproducts. Most recently, citrus peels were investigated for fortification of olive oil to analyze the phytochemical composition and sensory profile of the end product. The results showed that citrus oil had positive impacts on the sensory profile while enhancing the nutritional quality and acting as a guard on oxidative stress markers at the blood vessel level.^140^ Another study examined the possible utilization of orange byproducts in bakery manufacturing (brioches) as a fat replacer, and 50% of fat replacement with orange fibers allowed the production of brioches with improved technological and nutritional properties.^141^ Replacement of wheat flour in bread production by *Citrus* alpedo caused a higher perception of aftertaste despite of its high nutritional value.^142^ Therefore, it is important to optimize the formulations for the production of functional foods in order to limit the bitter aftertaste caused by citrus byproducts.

All in all, researchers have been focusing on the recovery of food industry wastes to maximize profits and minimize environmental impacts by the intention of extracting bioactive compounds. Specifically, increasing production capacities of the citrus industry has led to crucial amounts of waste generation, which contain a high quantity of health-promoting compounds. It can be concluded from these studies that different solvents and extraction parameters have a significant impact on the extraction yield of the bioactive compounds, which should be further studied for designing optimal conditions for specific sources and target extracts. Since the advantages of simultaneous application and green solvent utilization are evidenced, the establishment of larger scales for different industries should be considered in more detail. Moreover, suitable techniques should be further studied for the incorporation of these byproducts into food matrices to diminish the bitter aftertaste caused by the natural components of citrus fruits. Therefore, exploitation and integration of these bioactive molecules to foods and nutraceuticals requires further interdisciplinary research of toxicologists, chemists, sensory analysts, and food technologists.

### 2.3 Berries

#### 2.3.1 Grapes

Berries, especially grapes (*Vitis vinifera* L.), are one of the most cultivated fruit crops in the world and are widely processed for juice and wine. During grape processing, large amounts of solid wastes, namely, grape pomace and stems, are generated. Among these solid residues, only grape pomace itself accounts for almost 20% of all grapes processed, and they contain 4 to 5% phenolic compounds.^143^ Grape pomace contains large quantities of polyphenols including catechins, proanthocyanidins, and glycosylated flavonols, which act as free radical scavengers.^10^ Therefore, extracts of grape seeds and peels are increasingly gaining interest to produce functional food ingredients.^18^

In a study where different varieties of grapes were analyzed for their total phenolic content and proanthocyanidin contents, the Parellada cultivar yielded the greatest amount of total phenolics (4654 mg GA/100 g dw) and proanthocyanidins (92.1 mg tannin/g dw) among Chardonnay, Macabeu, and Premsal Blanc.^144^ In another study, grape juice pomace was reported as containing 59.6 mg GAE/g dw total phenolics and 96.93 mg tannin/g dw, which are similar to the previous research stated above.^10^ Authors have also analyzed the phenolic profile of grape juice wastes, and four different cultivars of grapes presented different phenolic profiles, including catechins, epicatechins, and some phenolic acids.^10,145^

Grape pomace contains significant amounts of total phenolics; however, grape pomace is not the only source of these health-promoting substances. Interestingly, grape stems were reported as a richer source of phenolics; almost twofold higher phenolics exist in stems compared to the pomace, depending on the cultivar, extraction method, and geography.^8^ There are various researches supporting this phenomenon. Llobera and Canellas (2006) have indicated that Manto Negro grape pomace obtained from the wine-making process contained 2.36 g GAE/100 g dw total phenolics, whereas 11.6 g GAE/100 g dw total phenolics was found in grape seeds.^146^ This variation between different parts of grape wastes originated from the production process, where some of the bioactives from grape pomace pass to the juice. On the other hand, stems are directly discarded and kept intact and, therefore, preserve their initial phenolic content.^143^

Novel extraction methods have also been reported recently to improve the extraction efficiency of grape pomace. In this sense, high hydrostatic pressure and enzyme-assisted extraction methods have been utilized in order to evaluate the most suitable combination of methods. Results have shown that high hydrostatic pressure enhanced the efficiency of enzymes up to 16 times during the extraction. On the other hand, enzymes had an inhibitory effect on the α-amylase activity. All in all, researchers have concluded that high hydrostatic pressure might be the more economical and efficient method for recovery of phenolics from grape pomace, compared to other sophisticated methods that require more time and energy for exactly the same procedure.^147^ A successful extraction of a polyphenolic fraction from cranberry pomace was also achieved through supercritical carbon dioxide and pressurized liquid extraction.^148^ Another study investigated the effects of ultrasound on the extraction efficiency of grape pomace, followed by encapsulation in an alginate-calcium matrix. Ultrasound-assisted extraction provided 1.4, 1.3, and 1.2 times higher antioxidant capacity, anthocyanins, and total phenolics, respectively. Besides, up to 29% of rutin and quercetin were detected, which implies that ultrasound is more efficient and feasible compared to conventional extraction. As for encapsulation, an encapsulated compound showed a greater stability in the absence of light. However, there is still need to evaluate the effects of different factors and establish a better matrix for the improvement of shelf life and utilization for functional foods.^149^ Recent studies on functional food production using grape pomace demonstrated that powdered grape pomace affected the textural properties of breadsticks; however, these products had acceptable sensory properties.^150^ Dried grape pomace was also evaluated in chocolate spreads, for the substitution of sugar and milk powders. All textural, rheological, and sensory parameters showed that grape pomace powder might be a good alternative for sugar and milk originated powders.^151^ It was also shown that 30% of the flour can be substituted by the grape pomace powder in sponge cake formulations.^152^ Pomace powders of chokeberries, bilberries, and elderberries have been also reported as good, inexpensive alternatives of food colorants, with wider food applications and a slower degradation of color.^153^ However, it is still necessary to understand the market needs in the food industry and establish new advertising strategies on sustainability and healthy lifestyle to enable the utilization of alternative food additives from industrial wastes. Additionally, recipe validation processes should be conducted to underline the threshold of acceptability, since these byproducts might be responsible for the astringent aftertaste after a certain level of usage.

#### 2.3.2 Other Berries

In addition to grapes, ∼8 million tons of strawberry (*Fragaria ananassa*) is being produced every year, and 7% of the manufactured strawberry ends up being a waste. Generally, the residual part of this fruit is being used as animal feed. However, a considerable amount of berry wastes are still being disposed to the landfills and contribute to the greenhouse gas (GHG) emissions because of the high organic load.^154^ Therefore, it is important for any waste to be evaluated in terms of generating new possibilities for both protecting the agricultural soil quality and taking advantage of finding inexpensive sources of bioactive substances.^155^ In this point of view, strawberry wastes are also valuable sources of phytochemicals such as tannins, phenolic acids, and flavonoids, and anthocyanin, the most abundant flavonoid, is responsible for the red color of berries.^154,156^ In strawberries, the most abundant anthocyanins were found as pelargonidin-3-glucoside, cyanidin-3-glucoside, 3-malonyl glucoside, and 3-rutinoside.^157^ Moreover, strawberry pulps are also rich in condensed tannins (up to 163 mg/100 g raw strawberry), which are also known as proanthocyanins, and the soluble phenolic content (SPC) of strawberry pomace was measured and found as 17 μg gallic acid equivalent/mg dw. In the same article, the SPC of black currant, red currant, blackberry, and raspberry was also discussed and reported as 50.44, 20.54, 46.50, and 36.72 μg GAE/mg dw, respectively.^154,157^

Strawberry achenes, although they occupy 1% of the total fruit, are responsible for 11% of the total antioxidant activity. This is mainly attributed to ellagitannins, ellagic acid, and its glycosides. Casuarictin, potentillin, and pedunculagin are identified.^158,159^ In a study where strawberry pulp was investigated for its total ellagic acid content, pulp with achenes exhibited a 6 times higher ellagic acid content compared to pulp without achenes.^160^ Another study has shown that achenes had the highest antifungal activity thanks to their high ellagic acid contents.^161^ Aaby et al.^162^ have reported that strawberry flesh contained 0.3 mg ellagic acid/100 g fw, whereas strawberry achenes contained 37 mg ellagic acid/100 g fw. Ellagitannins are mostly made up of ellagic acid, gallic acid, glucose, and hexahydroxydiphenoyl units gathered into a very similar structure. This brings up a problem of identification, where the mass spectra display almost the same patterns. As a consequence, correct identification of the ellagitannins from different sources is required to uncover the metabolism and bioactivity of these bioactive substances.^163^

Talking about black currant, the annual production reaches up to 185.000 tons worldwide, and almost more than half of the fruit ends up being pomace after processing.^164,165^ Although blackcurrant is a rich source of phenolic compounds (∼250 mg/100 g fresh fruit), its consumption is limited when it is fresh. Therefore, it is generally processed to produce juice, jams, and alcoholic beverages.^166^ As a result, large quantities of wastes are generated by blackcurrant juice processing, and unsurprisingly, considerable amounts of phytochemicals still exist in the byproducts.^167^ Blackcurrant wastes are also utilized in the industry for both producing functional food ingredients and pharmaceuticals due to its high amounts of bioactive compounds, including anthocyanins and phenolics. Therefore, berry wastes are no longer considered as waste, since there are myriad of opportunities to benefit from the high bioactive load of berries.^168^ The chemical composition of blackcurrant extract is given as 66.8 g/100 g total phenolics, 48.9 g/100 g total anthocyanins including delphinidin-3-rutinoside, delphinidin-3-glucoside, and cyanidin-3-rutinoside, in a decreasing order. Myricetin, quercetin, kaempferol, and their glycosides were reported as the dominant flavonol aglycones (13.3 g/100 g) and glycosides (4.6 g/100 g), respectively.^166^ In a similar study where an ultrasound-assisted extraction method was utilized to examine total phenolic compounds (TPC) and total flavonoid compounds (TFC) of blackcurrant pomace, chlorogenic acid was found as being the predominant phenolic (about 517 μg/g), followed by rutin (310.8 μg/g), naringenin (233.21 μg/g), and caffeic acid (10.472 μg/g).^169^ Considering the high bioactive content of blackcurrant, researchers have currently focused on valorization of berry pomace by introducing them into various food matrices to improve both nutritional and physical aspects.^170,171^

In addition to grapes, strawberries, and blackcurrant, other types of berries also contain a significant number of phenolic compounds. Blackberries are also rich sources of phenolic compounds and are consumed either as raw, minimally processed, or as processed foods such as juices, wine, tea, or jams.^172^ When processed, almost 20% of the fruit ends up being discarded as seed, peel, or stem and still contains some bioactive components.^173^ Blackberries contain significant amount of phenolic acids as gallic and caffeic acids, quercetin as a flavonol, and anthocyanins including cyanidin-3-glucoside, representing the 80% of the total anthocyanin content.^174,175^ The TPC of blackberry was reported as 4016.43 mg GAE/100 g dw, where the total monomeric anthocyanins were 364.53 mg cyanidin-3-glucoside/100 g dw. Caffeic acid is the most abundant phenolic compound with 55.87 mg/100 g dw, followed by myricetin with the concentration of 9.58 mg/100 g dw.^174^ However, depending on the extraction method and cultivation circumstances, compounds such as quercetin and kaempferol in residues were also reported in different studies.^176,177^ Blackberries also contain 11 different ellagitannins (pedunculagin, castalagin, vescalagin, Galloyl-HHDP glucose isomer, lambertianin C isomers, lambertianin A/Sanguiin H-6 isomers, lambertianin d isomer, galloyl-bis-HHDP glucose isomer, ellagic acid, and other unknown ellagitannis) in flesh, seeds, and torus, where most of them were concentrated in the seeds.^178^ Hager et al.^179^ reported that ellagitannins were quite stable when canned, pureed, and frozen. However, clarification of the blackberry juice caused up to 82% loss of ellagitannins due to the removal of ellagitannin-rich seeds. Therefore, it is implied that juice-processing conditions should be optimized to minimize ellagitannin losses caused by the removal of the seeds as presscake. On the one hand, even though high amounts of ellagitannins are present in blackberries, less than 1% of the ellagitannins (lambertianin A and C) were bioaccessible. On the other hand, almost 15% of ellagic acid was reported as being bioaccessible, due to its release from the breakdown of ellagitannins.^180^ This phenomena has led researchers to wonder about the correlation between blackberry consumption and possible health effects, concerning the bioaccessibility of the bioactive compounds. Ultimately, various studies present in the literature debate the positive effects of blackberries on cardiovascular diseases and specific types of cancers.^181−184^

Red berries also include blueberries, raspberries, red currants, and cranberries, which are also rich sources of phenolic compounds, mainly anthocyanins. Similar to other berries discussed above, these are also consumed both as fresh and processed into different products including juice. In this point of view, the total phenolic content and anthocyanin profile of blueberry, red raspberry, blackberry, and red currant pomaces were investigated by Jara-Palacios et al.^175^ They concluded that 11 different anthocyanins existed in blueberry pomace, mainly malvinidin-3-*O*-galactoside, malvinidin-3-*O*-arabinoside, delphinidin-3-*O*-galactoside, petunidin-3-*O*-galactoside, and residues of cyanidin derivatives. On the other hand, other berries contained only cyanidin derivatives, where cyanidin-3-*O*-glycoside existed in all pomace samples. In another study, a total of 15 anthocyanins was identified in the blueberry, and similarly, malvinidin-3-*O*-galactoside and malvinidin-3-*O*-arabinoside were reported as the most abundant anthocyanins, which account for 20 and 18% of total anthocyanins present in its structure, respectively.^185^ There are also similar reports on malvinidin glycosides being the major anthocyanins of blueberries, representing almost half of the anthocyanin profile of the fruit.^186,187^ As for red raspberries, Jara-Palacios et al.^175^ have found that cyanidin-3-*O*-sophoroside and cyanidin-3-*O*-glucoside were the dominant anthocyanins, representing 32 and 31% of the total anthocyanin content, and the former compound was not identified in other berries, which makes it a distinctive anthocyanin for red raspberries. Furthermore, red currants had the highest TPC (3446.59 mg GAE/100 g dw), followed by red raspberries (2014.66 mg GAE/100 g dw), blueberry (1954.54 mg GAE/100 g dw), and blackberry (1699.62 mg GAE/100 g dw) pomaces. Ellagitannins, such as dimeric sanguiin H-6, trimeric lambertianin C, monomeric casuarictin, potantillin, pedunculagin, sanguiin H-10 tetrameric lambertiainin D, and dimeric nobotanin A were also found in raspberries.^188,189^ Among these compounds, sanguiin H-6 was reported as being responsible for 45% of the free radical scavenging activity.^185,190^ Kähkönen et al.^190^ reported that 60% of the phenolic profile of red raspberries was made up of ellagitannins, 8% were of anthocyanins, and the remaining were of flavonols and phenolic acids. This research has also pointed out that ellagitannins may have inhibitory effects of oxidation of food emulsions for the first time. Therefore, it was concluded that ellagitannins might be utilized for the production of foods with high oxidative stability. Ellagic acids were also determined in cranberries, which tend to combine with flavan-3-ols to create ellagitannins in the presence of oxygen, heat, and light.^191^ There are various researches pointing out the high elagitannin content of cranberries.^192−194^

Even though berries are known for their rich antioxidant capacity, the sensitive nature of phenolic compounds causes a reduction of antioxidant activity during the digestion, mostly due to deviations in the pH and digestive enzymes.^195,196^ Therefore, a novel oral administration method is required in order to protect these bioactives against the harsh digestive environment that limits the bioavailability. A recent study examined the alginate and chitosan nanoparticles to establish an oral delivery system for grape pomace extracts by an ionic gelation method. The results suggested that both nanoparticles provided an improvement in the bioaccessibility of polyphenols as well as the antimicrobial and antioxidant effects of grape pomace extracts.^197^ Another study demonstrated that complexation of muscadine grape and blueberry phenolics with an edible protein fraction (rice-pea protein blend) to form aggregate particles, significantly enhanced their antioxidant and anti-inflammatory effects compared to the unmodified extracts.^198^

To sum up, different berries exhibit different qualitative and quantitative phenolic profiles regarding anthocyanins, phenolic acids, and flavonoids after they are processed into juice. Grapes show up as one of the most cultivated and processed berries and contain significant amounts of bioactive compounds, especially gallic acid and catechins. Strawberries are rich in anthocyanins, mainly in pelargonidins and tannins. Blackberries, blueberries, red raspberries, and red currant contain different varieties of anthocyanins in their structure. Therefore, it can be concluded that the food industry could use residues of berries as a good source of antioxidant compounds to obtain sustainable and cost-effective food ingredients with added value.

### 2.4 Stone Fruits

The stone fruits are characterized by a fleshy mesocarp (pulp) surrounding a wood-like endocarp or stone. The skin (epicarp) is thin and smooth except for peach and apricot, which have fine hair-like coatings. Stone fruit species include apricot, peach, nectarine plum (and greengage), cherry and they all belong to the genus Prunus.^199^ According to the recent FAO estimates (2019), the world production was nearly 4.1 million tons of apricots, 25.7 million tons of peaches and nectarines, and 1.4 million tons of sour cherries.^2^ These fruits, apricots, peaches, and sour cherries are main types of stone fruits that are processed into juice.^200^ As in other fruits, the main byproducts from the stone fruit juice industry are pomaces including seeds, skin, and pulp, which have high bioactive contents.^51^

Apricot pomace and kernel, which is a single seed found in stone, are the main byproducts including bioactive compounds such as polyphenols and fatty acids.^201^ Apricot pomace mainly consists of peel and pulp, which are the main byproducts from the apricot juice industry accounting for 40% of the total waste.^201,202^ It has been reported as a good source of β-carotene.^203^ Ruiz et al.^204^ reported that carotenoids in the peel are 2–3 times higher than in the flesh. In terms of the polyphenol composition of apricot byproducts, the current literature provides limited references.^205^ Among the present literature, Fan et al.^206^ reported phenolic compounds in the apricot peel to be ∼2–4 times higher than the pulp. Several biological activities of apricot kernels such as antimicrobial, antioxidant, and anticarcinogenic have been reported previously.^207,208^ These different activities make apricot kernels a potential compound for the medicine, cosmetic, and food industries. However, the use of kernels in the food industry is limited because they include amygdalin, which is converted to cyanide after ingestion. Cyanide is a compound also found in peach seeds, and it is reported to have the potential to be lethal in the 0.5–3.5 mg/kg body weight consumption doses.^209,210^ Looking at the antioxidant capacity and phenolic content, apricot kernels have been declared to have more antioxidant activity and phenolic content than the flesh.^211^ Qin et al.^212^ reported several flavones (e.g., apigenin 7-O-glucoside), flavanones (e.g., quercitrin and quercetin), anthocyanins (e.g., cyanidin-3-(4″-acetylrutinoside), and phenolic acids (e.g., salicylic acid and ferulic acid). Besides its polyphenol content, kernels are also rich in fatty acids. They comprise 40–50% of unsaturated fatty acids, which are mainly composed of oleic (60–70%) and linoleic acid (25–30%). The oleic and linoleic acid contents of apricot kernels were also reported to be 92 g/100 g of total fatty acids.^213^

Peach is a polyphenol-rich fruit, and these polyphenols are mainly localized in the pulp and peel tissues. The flesh and peel are rich in chlorogenic acid, neochlorogenic acid, proanthocyanidin B1, catechin, and epicatechin (proanthocyanidin monomers) as well as rutin, different glycosides of quercetin, and anthocyanins, which are also the primary phenolics in the peach fruit.^214,215^ Montevecchi et al.^216^ reported the amounts of catechin and chlorogenic acid in the pulp and peel of white flesh peach as 1160–2147 mg/kg in pulp and 1342–4578 mg/kg in peel. As with other fruits, peach peel is also richer (2–3 times) in total phenolic compounds than the flesh and the whole extracts.^217^ Contrary to these results, Loizzo et al.^218^ reported that the pulp contains more polyphenols and flavonoids than the peel. In addition, they also showed that the carotenoid concentration of peach peel was ∼6 times higher compared to seed and pulp. Anthocyanins are other bioactive compounds giving peach peel its red color, since they concentrate there. After the peel, the highest anthocyanin content is found in the stone,^219^ which is the other waste material obtained from peach processing. It contains a seed inside having a high protein content.^220^ Nowicka and Wojdyło (2018) studied the content of its bioactive compounds (polyphenols, carotenoids, and tetraterpenoids) and antioxidant activity. They reported the total polyphenols ranging between 12.7 and 3.8 g/100 g dw, carotenoids as 101.7–0.0 mg/100 g dw, and cyanogenic glycoside as 245.7–17.4 mg/100 g dw.^221^

The production of plum juice generates a large quantity of waste (pomace) as the skin and stone.^222^ As with peach and nectarine, plums are also rich in polyphenols; there are most of all flavonols and caffeic acid derivatives: neochlorogenic acid, chlorogenic acid, and cryptochlorogenic acid as well as anthocyanins and flavonols.^223^ The major polyphenol groups in plum pomace were reported as anthocyanins, proanthocyanidins, hydroxycinnamic acids, and quercetin glycosides.^223^ Like other fruits, the skin of the fruit was observed to have a three- to four-fold higher phenolic concentration compared to plum flesh.^224^ Wang et al.^225^ studied the purple Myrobalan plum fruit and skin. While the anthocyanins of the myrobalan plum peel varied from 1.93 to 19.86 g/kg, cyanidin-3-galactoside and cyanidin-3-glucoside were reported to be the most abundant anthocyanins. In addition, higher levels of anthocyanins and phenolics and higher antioxidant activity were reported in myrobalan plums than red and yellow types. In a study, proanthocyanidin content in Ruby sweet (blood plum) and Byron gold (yellow-fleshed) was compared, and the blood plum was observed to have higher proanthocyanidin content in both peel and flesh.^226^ The major polyphenol groups in plum pomace were reported as anthocyanins, proanthocyanidins, hydroxycinnamic acids, and quercetin glycosides.^223^ Savić et al.^227^ identified bioactive compounds in the plum seed extract such as amygdalin, gallic acid, vanillic acid, and benzoic acid. The plum seeds contain a high amount of oil, ∼30%, and include several bioactive compounds such as tocopherols, proteins, lipids, and phenolics.^227^

Sour cherry is an industrial fruit for which a high amount of waste is generated after processing. The byproduct of sour cherry comprises of pomace (skin and flesh) and seeds (pit, stone), which remain after the fruit juice. The pomace of sour cherry contains a high amount of phenolics, and its seed represents a high oil yield having positive effects on humans due to their antioxidant, antimicrobial, and anti-inflammatory properties.^228^ The total phenolic content of sour cherry pomace powder was reported to be 91.29 mg GAE/g dw.^229^ Yilmaz et al.^230^ reported the main phenolics in sour cherry pomace as neochlorogenic acid, cyanidin-3-glucosyl-rutinoside, and catechin. Demirdöven et al.^231^ reported the total anthocyanin, total phenolics, and antioxidant activity in plum pomace extracted with different methods. The total anthocyanin content (mg/L) ranged between 35.08 ± 1.06 and 38.20 ± 1.20, and the total phenolics were (mg/L) in the range of 453.27 ± 7.42 to 493.84 ± 5.12, whereas the antioxidant activity (mM Trolox/mL) was 59.61 ± 1.24 to 106.80 ± 0.71. Nowicka et al.^232^ reported the main phenolic compounds in dried sour cherries as polymeric proanthocyanidins that constituted 52.3% of the total phenolic compounds. In addition, the second most-abundant fraction was anthocyanin compounds (22.6%), especially cyanidin-3-glucosylrutinoside and cyanidin-3-*O*-rutinoside. The seeds of sour cherries contained a high amount of bioactive compounds such as essential fatty acids, carotenoids, sterols, and tocopherols.^210^ The oil content of sour cherries varies in different studies (17–36%).^233−235^ The seed was observed to be rich in unsaturated fatty acids, mainly oleic acid and linoleic acid.^210^

In recent years, studies on the use of environmentally friendly extraction technologies that reduce the use of organic solvents and energy consumption have been the focus. Therefore, many studies have been conducted on polyphenol recovery from different byproducts through emerging technologies such as infrared, ultrasound, and microwave-assisted extractions.^236^ In this regard, Cheaib et al.^236^ compared the efficiency of solid–liquid extraction (SLE), a conventional method, and three new extraction technologies such as ultrasound, microwave, and infrared (IRAE) in terms of polyphenol yield and bioactivity of apricot pomace. IRAE yielded the greatest polyphenol, flavonoid, and tannin contents, followed by MAE, UAE, and SLE. Furthermore, the strongest antimicrobial and antiradical activities were observed in infrared technology. Additionally, scanning electron microscopy (SEM) results showed that the IRAE technique caused the most cellular and structural damage in apricot pomace, which could be an explanation of the success of recovering polyphenols. More recently, Kasapoğlu et al.^237^ optimized the UAE parameters, time and temperature, with the aim of maximizing total phenolic and flavonoid contents as well as DPPH scavenging activity and extraction yield from apricot pomace. UAE performed at 50 °C, and 90 min trial points provided the best extraction yield, total phenolic and flavonoid contents, and DPPH scavenging activity. Moreover, *p*-coumaric acid, ferulic acid, and rutin were reported to be the most prevalent phenolic compounds in the extract. On the one hand, Plazzotta et al.^238^ compared the MAE and UAE for antioxidant compound extraction from peach waste and reported that both extraction methods for total phenolic, flavonoid, and anthocyanin compounds resulted in comparable yield percentages. On the other hand, vitamin C was successfully extracted by MAE only, due to the degradation during UAE. Furthermore, as compared to the UAE, MAE took half of the extraction time, less impactful on greenhouse gas emission, consumed less energy, and was more economically feasible when scaling up.

Recovery of bioactive compounds from agricultural wastes by recycling them to produce functional food products is of increasing interest, but the sensitivity of these compounds to external factors limits their use and bioavailability.^239^ In this context, an encapsulation method allows one to protect extracted bioactive compounds from environmental conditions and factors.^240^ Šaponjac et al.^241^ encapsulated the bioactive compounds recovered from cherry pomace in whey and soy proteins and then incorporated them in cookies by replacing flour. They followed the total polyphenols, anthocyanins, antioxidant activity, and color characteristics of cookies during four months of storage. The results of the study were promising for functional food development. No loss was observed in polyphenol content during the four months of storage, and antioxidant activity only showed a slight decrease, whereas the anthocyanin content significantly declined. In addition, enriched cookies showed acceptable sensory properties. Similarly, Oancea et al.^242^ encapsulated anthocyanins from sour cherry skin in whey protein isolate and incorporated them into fermented milk. Then, they evaluated their prebiotic effect on the probiotic strain. The coating compound preserved the anthocyanins from in vitro stomach digestion, allowing them to release into the intestine. During the storage period, the microcapsules were observed to also encourage the development of *Lactobacillus casei*. In addition, the color parameters revealed that the enriched milk has a reddish color, which could be an alternative natural colorant aside from functional properties.

Ultimately, stone fruits represent a large variety of byproducts from skin to the kernel skin found inside the stone. As in others, stone fruits possess high levels of bioactive components as mainly phenolics, and since the color of those fruits are mainly red (except for apricot), they are good sources of anthocyanins as well as proanthocyanidins. Currently, when the use of bioactive components in food products becomes much more important, extraction of anthocyanins and other bioactive components from the wastes of stone fruits would be advantageous. In addition to all these, encapsulation technology offers an efficient way to enrich foods and develop functional foods with better nutritional and sensory properties. However, the interaction of an encapsulated agent and coating material besides the food matrix is of critical importance. Therefore, future studies are required for the development of functional foods from encapsulated bioactive compounds.

### 2.5 Melons

Health benefits and the vital importance of fruits have already been discussed, as they contribute to overall health and potentially prevent some kinds of diseases including cancer. However, large quantities of bioactives are being lost during the production stages and discarded as a waste material. Melons are also a type of fruits that suffer from losing their valuable chemicals as a result of being processed into various kinds of end-products, mainly juices. *Cucumis melo L.*, also known as melon or cantaloupe, belongs to the *Cucurbitaceae* family, which also includes watermelon and honeydew. According to Agricultural Marketing Resource Center, ∼13 kg of melon per capita is being consumed every year in America.^243^ The relatively high consumption of these fruits can be attributed to their high organoleptic properties as well as nutritional attributes.^244^ Melon processing generates huge amounts of byproducts, mainly melon seeds, which are estimated as 738 tons according to FAOSTAT (2015).^2^ Mallek-Ayadi et al.^245^ analyzed the total phenolic content of melon seeds of the Maazoun cultivar, and they have reported the TPC as 304 mg/100 g, which is particularly important for preventing neurodegenerative and cardiovascular diseases, as proved by various studies.^246^ Among all phenolic compounds present in Maazoun cultivar seed oil, the most abundant phenolic acid was gallic acid (7.26 μg/g), followed by caffeic acid (3.13 μg/g), rosmarinic acid (2.91 μg/g), and protocatechuic acid (0.89 μg/g). Besides, amentoflavone as a flavonoid (32.80 μg/g) and some lignans were also identified (3.95 μg/g).^245^ Ismail et al.^247^ reported the TPC of cantoloupe seeds as 285 mg GAE/100 g extract, where total flavonoids content was 162 μg/100 g extract. The highest phenolic content was found in cantaloupe leaf as 2640 mg GAE/100 g extract, followed by the stem (1025 mg GAE/100 g extract), skin (470 mg GAE/100 g extract), seed, and flesh (168 mg GAE/100 g extract). Cantaloupe leaf was also the richest in total flavonoid content, 6970 μg/100 g extract. They also reported a high antioxidant activity in the leaf and stem of cantaloupe fruit. For honeydew seeds, Zeb^248^ reported 81.2 mg/100 g of total phenolics, which is much lower than that of others discussed above. When the phenolic profile was investigated, nine phenolics were identified, including caffeic acid, vanillic acid derivatives, quercetin-3-rutinoside, ellagitanins, derivatives of syringic, and ellagic acid. On the one hand, Galia melon seeds also contained lower amounts of total phenolics (57.2 mg catechin/100 g dw); on the other hand, watermelon seed is reported as a rich source of phenolics, ∼969.3 mg catechin/100 g dw.^249^ As for golden melon (aka Cucurmis melo var. inodorus Canary) seed, phenols, steroids, and flavonoids were the most abundant phytochemicals representing 29.93 mg/100 g, 22.63 mg/100 g, and 20.67 mg/100 g of the seed, respectively.^250^ Even though golden melon seed contain some bioactives, the amount is still below the other melon cultivars above. However, what we can conclude from these studies is melon seeds exhibit a good potential of extracting phytochemicals for valorization in different industries. In addition to phenolics, melon seed oil also exhibited similar properties of vegetable oils, including soybean and sunflower oils.^251,252^ Therefore, melon seeds may be great alternatives for the production of novel functional foods with a wide range of benefits.

Besides seeds, the juice, peel, and pulp of the Piel de Sapo cultivar (*Cucumis melo* var. inodorus H. Jacq.) were investigated with respect to their phenolics, carotenoids, and vitamin C content. The results showed that peels and seeds contained similar amounts of phenolics, and they presented the greatest values, 364 μg/g and 393 μg/g, respectively. On the other hand, the phenolic content of melon juice was found as 211 μg/g, and the pulp has 221 μg/g of phenolics, which is a valuable byproduct of juice processing. They also found similar values of vitamin C for juice, pulp, and peel, where seeds had lower values. Although there are some phenolics and vitamin C present, any type of carotenoid was not identified in Piel de Sapo melon.^253^ Unlike Piel de Sapo, cantaloupe melon contained 68.92 mg/g carotenoids in its pulp and 49.9 mg/g in its juice; however, total phenolics in the juice and the pulp was significantly lower than Piel de Sapo (95.35 μg/g and 101.9 μg/g, respectively).^244^

As for peels and seeds, they have contributed almost the half of the phenolics and antioxidant activity, and peel itself contained 31% of the TPC, which is comparable with the phenolics in juice (35%).^244^ Thanks to the high phenolic content of melons, peels are good sources of gallic acid, ellagic acid, and kaempferol along with other phenolics found in the seeds (ferulic acid, kaempferol, and gallic acid).^254^ Watermelon peel is reported to be the highest phenol-containing byproduct among different kinds of fruit peels including pawpaw, orange, pineapple, banana, apple, mango, and pomegranate,^1^ and the TPC of watermelon peels has been reported as 335 mg catechin/100 g dw by Duda-Chodak and Tarko (2007);^249^ it mainly contained gallic acid, catechin, ellagic acid, and kaempferol, which have been reported to have antiviral, antimutagenic, anticarcinogenic, and cytotoxic effects, along with a shade of cinnamic acid, ferrulic acid, chlorogenic acid, rutin, and some others.^255−257^

Various studies in the literature have evaluated the bioactive compounds in wastes of different melon cultivars, and they all agreed on the presence of valuable bioactive compounds, which are promising sources of natural food additives. However, there are limited studies on melon processing wastes; therefore, utilization of melon juice processing byproducts should be investigated in a deeper and more detailed sense to clarify possible market opportunities. There are some investigations on the encapsulation of melon extracts,^258−260^ but still their utilization in foods and pharmaceuticals is limited. For the utilization of melon byproducts for a sustainable food chain, it is necessary to evaluate different extraction techniques to establish a greener method for both the environmental issues and the public health. On the one hand, the validation of these methods is of the foremost importance in order to scale up to the industrial production. On the other hand, in vitro, in vivo, and shelf-life assessments should be performed in order to propose the possibilities of evidence-based functional food products.

### 2.6 Tropical Fruits

Tropical fruits are rich in nutrients such as vitamins (e.g., vitamins A, B, and C), minerals, and dietary fibers such as pectin, lignin, cellulose, and hemicellulose. Furthermore, some of the tropical fruits include high content of essential amino acids, proteins, and lipids. In addition to them, they also contain different types of secondary metabolites of which many are bioactive compounds. As stated before, these bioactive compounds impact human health in a positive way.^261^ According to FAO estimates (2019), the world production of mango is estimated to be greater than 50 million tons, while that of pineapple is nearly 30 million tons and that of kiwi fruit is higher than 4 million tons.^2^

Mango peels have gained much attention in recent years, since it possesses valuable components including phytochemicals, carotenoids, polyphenols, enzymes, and vitamins E and C, all of which have functional and antioxidant properties.^262^ Nowadays, the functional food industry uses mango peel flour in several products such as bread, biscuits, sponge cakes, and noodles.^263^ Jiang et al.^6^ isolated the two major compounds, namely, ethyl gallate and penta-*O*-galloyl-glucoside, from mango peels. They observed that these compounds present potent hydroxyl radical, superoxide anion, and singlet oxygen scavenging activities.^6^ Like mango peels, mango seeds are also byproducts of mango processing. The kernel comprises 45–85% of the seed and ∼20% of the whole fruit depending on the varieties.^264^ Maisuthisakul and Gordon^265^ studied the antioxidant and tyrosinase inhibitory properties of extracts from mango seed kernels. They observed that extracts of mango seed kernel have phenolic components having high antioxidant activity as well as tyrosinase inhibitory activity. The extracts were also reported to have the most efficacious antioxidant with the highest metal-chelating, radical scavenging, and tyrosinase inhibitory activity. Berardini et al.^266^ reported the gallotannins, which is a compound having high antioxidant and significant antiproliferative activity, in mango peel and kernels as 4 and 15.5 mg/g dw, respectively. Consistent with this study, Luo et al.^267^ also reported that mango kernels contain more gallotanins compared to its peel. The authors declared that gallotannin-rich extracts from mango kernel and mango peel could be a potential source of anticancer agents. TPC in the peel and seed kernels of the Ubá variety of mango fruit was evaluated by Ribeiro et al.^268^ The results showed that mango seed kernels were richer in total phenolics than the peel. Sogi et al.^269^ reported the amounts of total phenolics (mg/100 g), ascorbic acid (mg/100 g), and carotenoids (μg/100 g) in dried mango peels as 2032–3185, 68.49–84.74, and 1880–4050, respectively, and in powdered mango kernels as 11 228–20 034, 61.22–74.48, and 370–790, respectively.

The industrial processing of pineapple, including minimal processing, generates a high quantity of byproducts, which generally represent more than 20% of the fruit.^270^ The main byproducts of pineapple are core and rind, and these byproducts represent 25–35% of the pineapple fruit; the rind is the predominant one.^270,271^ Looking at the rind, Larrauri et al.^271^ reported myricetin, salicylic acid, tannic acid, *trans*-cinnamic acid, and *p*-coumaric acid identified in a high dietary fiber powder from pineapple rind as potent antioxidants. Guo et al.^272^ reported the phenolic antioxidants in pineapple peel as 2.01 mmol /100 g fw. More recently, polyphenolic metabolites from pineapple peel such as gallic acid, catechin, epicatechin, and ferulic acid, which participated in the reduction of oxidative stress-related diseases, were identified.^273^ Bromelain, a protease, is found in pineapple stem, core, peel, and crown.^274^ Bromelain extracted from pineapple core was reported to be 26 kDa by Hebbar et al.^275^ Looking at the stems, Maurer^276^ reported that the bromelain extracted from stems was 23.8 kDa. In terms of ascorbic acid and carotenoid content, Freitas et al.^270^ reported that pineapple core has ∼2 times higher vitamin C content, while the rind has ∼2.5 times higher β-carotene.

Kiwi pomace, which consists of a heterogeneous mixture of skins, seeds, calyx, and pulp,^277^ is the primary byproduct from the kiwi juice industry, and it accounts for 20–40% of the whole fruit.^278^ There is limited information in the literature on the exploitation of kiwi fruit byproducts such as seeds or peels. Therefore, the bioactive compounds and antioxidant properties of various parts of the kiwi fruit have not been well-investigated. Zhu et al.^277^ reported TP in kiwi pomace (skin not included) as 421 ± 12 mg GAE/g dw. They did not observe anthocyanins, since the kiwi fruit is not red. Furthermore, flavan-3-ol also was not determined. They reported the major phenolic compounds in kiwi pomace as quinic acid, caffeic acid, and its derivatives, followed by kaempferol derivatives and a small amount of quercetin derivatives. Kheirkhah et al.^279^ also reported the most abundant phenolic compounds in the kiwi pomace, which were catechin, chlorogenic acid, *p*-coumaric acid, protocatechuic acid, and caffeic acid.^279^ Soquetta et al.^280^ produced flour from the green kiwi fruit skin of two varieties (Bruno and Monty). The flour made from the Bruno variety showed higher DPPH values and levels of phenolic compounds (1262.34 mg GAE/100 g flour), while the Monty variety had higher FRAP values, vitamin C (189.06 mg/100 g flour), flavonoids (486.47 mg/100 g flour), chlorophylls (12.13 mg/100 g flour), and carotenoids (246.91 g/100 g flour). Deng et al.^281^ reported that the DPPH activity of the seeds of different species of kiwi fruit was reported to change from 25.7 to 35.1 IC_50_ mg/mL. Salama et al.^282^ also studied the same species, but their study was on the in vitro antioxidant activity in peel, which has been reported as 21–47 μM TE/g. Duman and Özcan^283^ reported the major fatty acids in kiwi seed as palmitic, oleic, and linoleic acids. Wang et al.^284^ reported the phenolic compunds in peel, seed, and flesh. In seed, the major phenolics in different cultivars were caffeic acid, quercetin, catechin, *p*-hydroxybenzoic acid, and *p*-coumaric acid.

Pomegranate peel accounts for 50% of the fruit, which is rich in several compounds including phenolics, flavonoids, ellagitannins, and proanthocyanidin compounds.^285^ Punicalagin and punicalin, both of which include the polyphenolic chemical component gallagic acid, the building block for many tannins, are ellagitannins found in the pomegranate peel. Punicalagin is a pomegranate-specific compound found in the seeds, peels, and juice.^286^ Actually, the content of tannins and flavonoids is mostly linked to biological characteristics of pomegranate peel. The peel of the fruit has the strongest antioxidant activity, which is consistent with its high polyphenol concentration.^272,285^ Surveswaran et al.^287^ reported that peels represented high total antioxidant capacity, while seed had a lower value. Similarly, Hajimahmoodi et al.^288^ reported that, among peel, pulp, and seeds, the peel showed much higher antioxidant activity. Moreover, pomegranate peels also showed a higher antioxidant capacity in vitro when compared to other fruits such as mangos, bananas, and coconuts.^289^ The seeds of the pomegranate have low polyphenolic content and antioxidant activity.^272^ However, it is rich in oils and includes a unique fatty acid profile characterized by a high content of fatty acids such as linoleic acid and linolenic acid.^286^ Özgül-Yücel^290^ studied the conjugated fatty acid content of oil-containing foods such as pomegranate, mahaleb, catalpa, and oregon grape. Pomegranate seed was the one that contained the highest amount of conjugated linoleic acid. Sadeghi et al.^291^ determined the FRAP value of the seed fraction of six different cultivars of pomegranate. They reported that the antioxidant activity varies among the cultivars and that the Sour white peel had the highest FRAP value.

Bioactive compounds have been recovered from tropical fruit byproducts by applying a variety of novel extraction processes.^292^ Among the novel technologies, the use of green solvents, which reduce the negative impacts of the use of solvents on the environment, has emerged.^293,294^ Supercritical fluids, ionic liquids, and deep eutectic solvents have been the most actively investigated as possible green solvents in the last two decades, particularly in the fields of food and medicinal plant processing.^295^ Deep eutectic solvents provide a higher yield of extraction from natural compounds than those obtained from conventional methods. In addition, they offer an inexpensive, recyclable, nontoxic, and environmentally friendly solvent.^296,297^ Bhushan et al.^296^ investigated the recovery of polyphenolic compounds from ripe mango peel using deep eutectic solvents based on a microwave-assisted extraction technique. The results revealed that, under optimized conditions (436.45 W power, 59.82 mL/g liquid-to-solid ratio, in 19.66 min), the total phenolic content was 56.17 mg GAE/g dw. A deep eutectic solvent system composed of lactic acid/sodium acetate provided the best yield for total phenolic content from mango peel. In addition, mangiferin was reported as the prominent phenolic compound in the mango peel extracts. Sánchez-Mesa et al.^298^ extracted the bioactive compounds from mango peel by PLE and SFE. As a cosolvent, ethanol was used for SFE and water for PLE. The results showed that, even though PLE provided better yields, a higher concentration of compounds such as gallotanins and flavonoids was obtained by SFE. Because of the enhanced solubility of the compounds in ethanol with increasing percentages, SFE would promote the recoveries at higher concentrations.

Waste generated from tropical fruits can be minimized by the development of value-added products.^299^ Chappalwar et al.^300^ developed functional chicken patties with the incorporation of mango peel powder as a fat replacer. Then, they evaluated the product for its physicochemical properties and sensory attributes. The results indicated that, while emulsion pH, emulsion stability, fat and cholesterol contents, and water activity of mango peel-treated chicken patties were significantly lower, the moisture content, cooking yield, fat, and moisture retention values were significantly higher than that of the control. However, sensory scores were decreased by mango peel powder incorporation. Fruit waste byproducts were not only used in functional foods but also in edible film production. Rojas-Bravo et al.^301^ developed a starch edible film by incorporating mango peel powder and evaluated the effect of its addition on physical, structural, and antioxidant properties as well as their effectiveness as edible coatings on apple slices during storage (4 °C). Mango peel powder applied as edible coating increased the firmness, browning index (BI), total phenolic content, and antioxidant capacity of apple slices. In addition, edible coating application decreased the firmness of apple slices during storage. In this context, the application of fruit wastes on edible film packaging should further be studied and optimized.

In short, tropical fruits include different bioactive components, and the byproducts from the juices manufactured from tropical fruits have the potential to be a good source of different bioactive components. In this sense, it would be rewarding to give the necessary importance to the bioactive wastes of these fruits.

## 3 Conclusion and Future Perspectives

Fruits have been consumed either raw or processed since the beginning of the world. Currently, the demand for fruits and vegetables has increased due to several reasons including the growing population and people’s choices to consume healthier foods. The shortage of raw materials for the food industry brings up environmental, economic, and social problems, which ends up causing an increase in the rise in the costs of food products; therefore, the number of people suffering from extreme hunger is ever-increasing. In this regard, the valorization of byproducts obtained in the food industry and the development of new functional products attract great attention in terms of providing a significant economic return in the industrial dimension. Furthermore, from the perspective of possible future food shortages, the importance of waste assessment emerges as an indisputable fact. Every year, a huge number and amount of byproducts are formed among the various branches of the food industry, especially in fruit processing. Currently, fruits are processed into several products including fruit juices, which accounts for 50% of the crops that are being processed. Apples, citrus fruits, berries, stone fruits such as apricot and peach, melons, and tropical fruits constitute the majority of the fruit juice industry. The production of such juices generates a high quantity of byproducts that include mainly peels, seeds, pulp, and stems. These wastes may possess a variety of bioactive compounds that are known to have several beneficial effects on human health. The fact that these products have bioactive substances with various biological activities makes it possible to use them in food, pharmaceutical, and cosmetic industries. There are several studies in the literature on the valorization of fruit waste in different areas, and the variety of studies is increasing exponentially every day. However, the high cost of the process may limit some researchers. In this context, research studies should be supported especially to ensure higher-efficiency extraction and valorization of fruit wastes.

Conventional extraction methods are generally unfavorable, since they require a high expenditure of energy and time as well as the utilization of organic solvents, which are hazardous to the environment and to human health. In contrast, novel technologies have been regarded as more advantageous than conventional methods; however, the method should be selected and implemented wisely by taking the food matrix and target compound into consideration. Mostly, the combination of different techniques and pretreatments is expected to be more practical to increase the extraction yield. Optimization of the extraction method and conditions may be of critical importance to be able to provide the maximum yield with the best quality of products and needs in-depth investigations for each individual fruit juice waste considering their different profiles and contents of bioactives.

However, the production of novel food ingredients at larger scales might be compelling in the technological and economical point of view. Waste valorization could be a good option by taking advantage of finding inexpensive sources of bioactive substances; however, it should be also considered that implementation of a new infrastructure (i.e., drying units) will be required to safely process wastes at an industrial scale, which means additional investment costs. In this case, governmental supports come to the fore more, and their importance increases. These supports will encourage investors and researchers, and it will be a good investment for the near future.

Another important issue to be considered in future studies is the food application potential of fruit juice byproducts. In the literature, there are limited examples for the application of these byproducts as a fat replacer, sugar replacer, colorant, or added to improve the nutritional value of the foods. However, the number of these applications needs to be improved also considering the applications at a commercial level. Besides, the bioaccessibility and bioavailability characteristics of the bioactive compounds obtained from fruit wastes as well as the functional foods prepared by using these wastes/bioactives need to be explored. During these food applications, the reactions of the consumers as well as the sensory attributes of the final products should be also considered.

Encapsulation methods to improve the stability and shelf life of these bioactives might be promising to improve their application potential in food and pharmacology purposes. Although there are various studies on the encapsulation methods and their consequences on fruit bioactives, a specific regulation should be legislated for the safety assessments of the bioactives from byproducts to prevent valorization of potentially hazardous compounds. It is expected that the importance given to bioactive substances in fruit wastes will increase in the near future and that it will contribute greatly to sustainability and circular economy. However, several concerns including safety issues, toxicological aspects, consumer acceptance, and sensory attributes of the formulated foods including extracts of fruit wastes should be also considered in order to develop evidence-based functional foods.
